# Nisin improves the storage quality of refrigerated pre-packaged fermented soybean whey-tofu—food safety, texture, and flavor

**DOI:** 10.3389/fnut.2026.1807620

**Published:** 2026-03-30

**Authors:** Jie Wu, Wen Zeng, Shucheng Liu, Hao Chen, Jianghao Wu, Liuyu Wei, Chengjun He, Dan Zhao, Zhanrui Huang, Jianrong Wang

**Affiliations:** 1 Hunan Provincial Key Laboratory of Soybean Products Processing and Safety Control, Hunan Engineering Research Center of Green Processing and Equipment of Hunan-Style Food, College of Food Science and Chemical Engineering, Soybean Products Industry Research Institute, Shaoyang University, Shaoyang, China; 2 Guangdong Provincial Key Laboratory of Aquatic Product Processing and Safety, College of Food Science and Technology, Guangdong Ocean University, Zhanjiang, China; 3 Hunan Xianrun Biotechnology Co., Ltd., Shaoyang, China

**Keywords:** biogenic amines, fermented soybean whey-based tofu, flavour, nisin, storage quality

## Abstract

**Introduction:**

Fermented soybean whey-based tofu, a traditional and popular food product, faces significant spoilage issues during storage, including texture softening, elevated volatile basic nitrogen (TVB-N) levels, and accumulation of biogenic amines, which can compromise sensory attributes and pose health risks. Current preservation strategies, such as physical sterilization and chemical preservatives, have limitations due to potential nutritional loss and safety concerns. Therefore, there is an urgent need for safe, efficient, and eco-friendly preservation technologies. This study evaluates the potential of nisin, a natural bacteriocin, as a preservative for fermented soybean whey-based tofu.

**Methods:**

Fermented soybean whey-based tofu was treated with different concentrations of nisin (0, 0.5, 1, and 2%) and stored at 10 °C for 28 days. The effects of nisin on physicochemical properties, biogenic amine accumulation, sensory attributes, and volatile flavor profiles were assessed.

**Results:**

Nisin significantly inhibited microbial growth, reduced biogenic amine accumulation (histamine and tyramine) by 40–60%, and maintained water-holding capacity and sensory scores. The 2.0% nisin treatment showed the best preservation effect, but 1.0% was deemed most suitable considering market acceptance and cost. Nisin also suppressed off-flavor compounds while retaining key aroma contributors, as confirmed by electronic nose and gas chromatography–ion mobility spectrometry (GC–IMS)/gas chromatography–mass spectrometry (GC–MS) analyses.

**Conclusion:**

Overall, nisin effectively enhanced the storage quality and shelf life of fermented soybean whey-based tofu through multidimensional mechanisms, offering a natural preservation strategy for traditional soy products.

## 1 Introduction

Societal advances have heightened consumer concerns about the safety and salubrity of food products. This has precipitated a marked increase in demand for clean label and natural food preservatives. The development of bioactive peptide-based preservation technologies has emerged as a frontier research focus in food science in response to this trend (1). Nisin, a bacteriocin naturally produced by certain strains of *Lactococcus lactis*, exhibits high efficacy, biosafety, and biodegradability (2). Nisin demonstrates selective inhibition against Gram-positive bacteria, showcasing significant potential in food preservation (3). Its application has been extensively validated in the dairy and meat product industries (4, 5), where it effectively extends shelf life while maintaining product quality. However, the complexity of food systems results in divergent mechanistic actions of nisin across different food matrices (6). In plant-based protein matrices, particularly widely consumed traditional foods such as tofu, the multidimensional quality control mechanisms mediated by nisin are under-researched (7). While extant studies have partially elucidated its antimicrobial effects, the synergistic interactions of nisin in suppressing biogenic amine formation, maintaining gel network stability, and modulating flavor profiles have not been comprehensively investigated (8).

Fermented soybean whey-based tofu (acid whey-coagulated tofu), a representative traditional Chinese non-fermented soy product, has gained widespread consumer popularity due to its characteristic flavor profile and textural properties (9). In recent years, tofu consumption has increased steadily, surpassing 8 million tons in 2025 (10). However, the dual effects of microbial contamination and enzymatic reactions result in a series of quality deterioration issues in fermented soybean whey-based tofu, including textural softening, elevated volatile basic nitrogen (TVB-N) levels, and biogenic amine accumulation (11). These issues can potentially compromise the sensory attributes and product quality of fermented soybean whey-based tofu and pose potential health risks to consumers.

Current preservation strategies for fermented soybean whey-based tofu predominantly involve physical sterilization and the use of chemical preservatives (12). However, physical sterilization (e.g., high-temperature treatment) can degrade heat-sensitive nutrients such as certain vitamins (e.g., B vitamins) and proteins, potentially reducing the overall nutritional value of the product. Furthermore, chemical preservatives have raised safety concerns due to residue risks, thereby intensifying consumer apprehensions about food safety (13). Consequently, developing safe, efficient, and eco-friendly preservation technologies has become imperative for the fermented soybean whey-based tofu industry.

This study innovatively applied nisin to pre-packaged fermented soybean whey-based tofu systems, demonstrating its significant efficacy in enhancing storage quality through multidimensional synergistic regulation. Nisin effectively inhibited microbial proliferation, delayed quality deterioration, and preserved favorable sensory attributes and flavor profiles. The findings of this study provide a scientific foundation for the application of nisin in the preservation of fermented soybean whey-based tofu, offering critical theoretical insights and practical implications to advance modernization strategies for traditional soy-based food production. Notably, this study integrates sensory analysis, texture testing, electronic nose, gas chromatography–ion mobility spectrometry (GC–IMS), and gas chromatography–mass spectrometry (GC–MS) to offer a rare, multi-layered perspective on tofu quality during storage. This comprehensive methodological integration is a strength of the study, as it provides a more holistic understanding of the effects of nisin on tofu quality compared to previous studies that focused on limited parameters. This study evaluates nisin’s preservation effects under controlled refrigeration (10 °C), with a focus on physicochemical and sensory properties. Future studies may expand to variable storage conditions, microbial community profiling, and combined preservation methods.

## 2 Materials and methods

### 2.1 Materials

Non-GMO soybeans (protein content 40%) were supplied by Jinzai Food Group Co., Ltd. All soybeans used in this study were from the same batch to ensure consistency. Nisin was from Zhejiang Xinyinxiang Biotechnology Co., Ltd. The nisin used in this study was of analytical grade with a purity of 99.5%, ensuring high consistency and reliability in the experimental results. All other chemicals were obtained from commercial sources and were of analytical grade.

### 2.2 Sample preparation

Fermented soybean whey-based tofu was prepared using the method described by Huang et al. (9). The tofu was cut into blocks (250 g; 7.0 × 10.0 × 4.0 cm) and aseptically packaged into sterile pre-packaged containers. The samples were randomly divided into four groups, with each container supplemented with approximately 90 g of immersion solution containing 0% (control group, CK), 0.5% (nisin 0.5% group), 1% (nisin 1% group; this was the experimental group for GC–MS and GC–IMS), or 2% (nisin 2% group) nisin. The packages were heat-sealed (185 °C for 25 s), subjected to reverse-pressure sterilization (85 °C for 30 min), and stored at 10 °C for 28 days. Quality indicators were analyzed at predetermined intervals (0, 4, 8, 12, 16, 20, 24, and 28 days) during storage.

The storage conditions were carefully controlled to ensure consistency across the study. The storage temperature was maintained at 10 °C ± 0.5 °C using a temperature-controlled incubator. Humidity was monitored and maintained at 60 ± 5% relative humidity by placing a hygrometer inside the incubator. Light exposure was minimized by storing the samples in a dark room with no direct sunlight. Storage conditions were recorded daily to ensure they remained within the specified range throughout the study.

### 2.3 Physical and chemical determination

#### 2.3.1 Water-holding capacity

A predetermined mass (*m*₁) of fermented soybean whey-based tofu was transferred into a centrifuge tube and centrifuged at 4,000 rpm for 15 min to remove surface moisture. After drying with filter paper, each sample was reweighed to determine the post-treatment mass (*m*₂). The water-holding capacity (WHC) was calculated as follows:

WHC(%)=(m₂/m₁)×100%.

#### 2.3.2 Moisture content determination

Samples were accurately weighed (denoted as *m*₁), placed in an oven at 105 °C, and dried to constant weight (*m*₂). The moisture content was calculated as:

Moisturecontent(%)=(m₁−m₂)/m₁×100%.

#### 2.3.3 Total acidity determination

A predetermined mass of fermented soybean whey-based tofu was homogenized with deionized water and filtered. The filtrate was titrated with NaOH (0.1 M) standard solution using phenolphthalein as the indicator until a faint pink endpoint persisted for 30 s. The total acidity, expressed as lactic acid equivalent, was calculated as:

Total acidity(%)=(c×V×0.090)/m×100%.

Here, *c* is the NaOH concentration (M), *V* is the consumed NaOH volume (mL), *m* is the sample mass (g), and 0.090 represents the millimolar mass of lactic acid (g mmol^−1^).

#### 2.3.4 pH determination

A predetermined mass of tofu sample was homogenized with deionized water. The pH (PHB-5, Inesa Scientific Instrument Co., Ltd., China) probe was immersed in the homogenate, and the pH value was recorded when the reading stabilized.

#### 2.3.5 Total viable counts

Tofu samples (25 g) were serially diluted, and 1 mL aliquots were transferred to sterile Petri dishes, mixed with nutrient agar medium, cooled to approximately 46 °C, and allowed to solidify. The plates were inverted and incubated at 37 °C for 48 h. Colonies were enumerated, and the total viable count was calculated as colony-forming units per gram (CFU g^−1^) based on the dilution factor.

#### 2.3.6 Malondialdehyde (MDA) determination

The determination was carried out using the method of Dai et al. (7). Tofu samples were homogenized and mixed with thiobarbituric acid (TBA) solution. The mixture was heated in a boiling water bath, cooled, and centrifuged. The supernatant absorbance was measured at 532 nm. The MDA content was calculated using a standard curve, which was generated by reacting TBA with MDA standards of various concentrations and measuring the absorbance of the solution at 532 nm.

#### 2.3.7 Total volatile basic nitrogen (TVB-N) determination

The determination was carried out using the method of Dai et al. (7). Tofu samples were mixed with magnesium oxide suspension and subjected to steam distillation to release VB-N. The liberated VB-N was collected in a boric acid absorption solution and titrated with standardized hydrochloric acid (HCl). The TVB-N content was calculated as follows:

$$\mathrm{TVB}-N\;\left(\mathrm{mg}100g^{-1}\right)=\left(V_{1}-V_{2}\right)\times c\times 14\times 100/m.$$

Here, *V*_1_ = HCl volume consumed for sample titration (mL), *V*_2_ = blank titration volume (mL), *c* = HCl concentration (M), *m* = sample mass (g), and 14 = nitrogen molar mass (g mol^−1^).

### 2.4 Bioamine assay

The method of Zhang et al. (14) was followed with modifications. Specifically, the concentration of the trichloroacetic acid solution was adjusted from the original 10% (v/v) to 5% (v/v) to better suit the sample matrix and improve extraction efficiency. The ultrasonic extraction time was extended from 20 to 30 min to ensure more complete extraction of biogenic amines from the tofu matrix. Additionally, the reaction time for the derivatization step was increased from 30 to 60 min to ensure complete reaction of biogenic amines with dansyl chloride, thereby enhancing the detection sensitivity. Trichloroacetic acid solution (5%, 10 mL) was added to the tofu sample (5 g). The mixture was homogenized, ultrasonically extracted for 30 min, and centrifuged at 10,000 rpm for 15 min. The supernatant was collected and filtered through a 0.45 μm microporous membrane. The derivatization reaction was then performed. The filtered supernatant (1 mL), dansyl chloride acetonitrile solution (0.1 mmol/L, 100 μL), and sodium bicarbonate solution (0.2 mmol/L, 100 μL) were mixed well and reacted in a 60 °C water bath in the dark for 60 min. After the reaction was complete, ammonia water (2 mmol/L, 50 μL) was added, and the mixture was shaken for 10 min to terminate the reaction. Then n-hexane (1 mL) was added, and the mixture was shaken for 10 min. The upper n-hexane layer was discarded, and the lower aqueous phase was filtered through a 0.22 μm microporous membrane for HPLC (UltiMate 3000, Thermo Scientific, USA) analysis. HPLC analysis conditions: C18 chromatographic column (250 mm × 4.6 mm, 5 μm); mobile phase A, acetonitrile; mobile phase B, ammonium acetate solution (pH 4.5, 0.05 mmol/L), with a gradient elution program; flow rate, 1.0 mL min^−1^; column temperature, 30 °C; detection wavelength, 254 nm; injection volume, 20 μL. Qualitative and quantitative analyses were performed based on the retention times and peak areas of the biogenic amine standards, and the content of biogenic amines in the sample was calculated using the external standard method. The calibration curves for each biogenic amine standard showed good linearity (*R*^2^ > 0.99) over the tested concentration range.

### 2.5 Sensory evaluation

A review panel comprising 10 trained evaluators from Shaoyang University (5 males and 5 females, aged 20–50 years, mean age 28 years) participated in the evaluation. Prior to the test, all panelists underwent 6 h of training in assessing sensory attributes, including appearance, color, structure, and odor. Each 20 g sample was placed in a coded transparent vial, assigned a three-digit random code, and presented in random order. Each sample was evaluated 3 times. The evaluation criteria were based on a ten-point scale:

Appearance: The smoothness and integrity of the tofu surface were observed. If the surface was smooth with no apparent cracks or dents, it scored 8–10 points; if there were slight defects, it scored 5–7 points; if the surface was rough and there were many defects, it scored 1–4 points.

Color: The original color and uniformity of the tofu were observed. If the color was natural and uniform, it scored 8–10 points; if the color was slightly altered but did not affect the overall sensory experience, it scored 5–7 points; if the color was abnormal and dull, it scored 1–4 points.

Structure: The texture and firmness of the tofu were determined by pressing with the fingers. If the texture was compact and elastic, it scored 8–10 points; if the texture was slightly soft but still acceptable, it scored 5–7 points; if the texture was loose and easily broken, it scored 1–4 points.

Odor: The smell of the tofu was observed. If it had a typical soybean smell and no peculiar smell, it scored 8–10 points; if there was a slight peculiar smell but it was not pungent, it scored 5–7 points; if there were obvious odors such as rancidity and putrefaction, it scored 1–4 points. Finally, the average of the sensory evaluation scores from all assessors was calculated.

Limiting values of acceptability: A minimum score of 5 points for each attribute (appearance, color, structure, and odor) was set as the threshold for acceptability. Samples scoring below 5 points in any attribute were considered unacceptable for consumption. The average score of all evaluators was calculated, and samples with an overall score below 5 were deemed unsuitable for market distribution. The study was conducted in accordance with the guidelines of the Declaration of Helsinki and approved by the Biomedical Research Ethics Committee of Shaoyang University. All participants provided informed consent and retained the right to withdraw from the sensory assessment at any stage (approval number: 2023KYKT077).

### 2.6 Texture test

The texture of the tofu was measured using a texture analyzer (TA1, LLOYD, UK) with a three-point sampling method to determine its hardness, springiness, chewiness, and resilience. The testing conditions were as follows: Texture profile analysis (TPA) mode, P35 cylindrical flat probe, pre-test force 0.05 N, compression speed 60 mm min^−1^, post-test retract speed 50 mm min^−1^, inter-test interval 5 s.

### 2.7 Electronic nose measurement

Electronic nose measurements were performed using an electronic nose system (Shanghai Angshen Intelligent Sensory Technology Co., Ltd., Changsha, China). The sample was chopped, and 5 g was placed in a 30 mL sampling vial, which was equilibrated in a water bath at 50 °C for 5 min before testing. The parameters were as follows: sampling interval, 1 s; cleaning time, 120 s; pre-sampling time, 10 s; detection time, 120 s; flow rate in the sensor chamber, 300 mL min^−1^; sample flow, 300 mL min^−1^. The information on the electronic nose sensor is shown in Supplementary Table S1.

### 2.8 Headspace-gas chromatography–ion mobility spectroscopy (HS-GC–IMS) volatile flavor compound analysis

A gas chromatography-ion mobility spectrometer (FlavourSpec 1 H1-100053, GAS, Germany) with an Rtx-wax column (30 m × 0.53 mm, 1 μm; Restek, USA) and an automated headspace injection unit (Combi PAL; CTC, Switzerland) was used in this study.

Headspace sampling was used, and the sampling conditions were optimized. The optimized headspace sampling conditions were as follows: 0.5 mL of the sample was sealed in a 20 mL headspace vial, heated and shaken at 70 °C for 15 min, and then 200 μL of the sample was taken from the upper space of the headspace vial for analysis. The temperature of the headspace injection needle was 85 °C. The GC–IMS test conditions were as follows: temperature of the GC column, 60 °C; temperature of the drift tube, 45 °C; flow rate of the carrier gas for GC–IMS, 2 mL min–1 for 0–2 min, 2–10 mL min^−1^ for 2–10 min, 10–90 mL min^−1^ for 10–30 min and 90 mL min^−1^ for 30–40 min; IMS drift gas flow rate, 150 mL min^−1^. Both carrier gas and drift gas were nitrogen (purity: 99.999%). Each sample was prepared in triplicate.

### 2.9 Headspace-gas chromatography–mass spectroscopy (HS-GC–MS) volatile flavor compound analysis

Tofu sample (5 g) was placed in a 15 mL headspace vial. Sodium chloride (2 g) was added, and a 50/30 μm divinylbenzene/carboxen/polydimethylsiloxane (DVB/CAR/PDMS) extraction fiber was inserted. Headspace extraction was performed in a 60 °C water bath for 30 min. After the extraction was complete, the extraction fiber was inserted into the GC–MS injection port and desorbed at 250 °C for 5 min.

For GC, a DB-5MS capillary column (30 m × 0.25 mm × 0.25 μm) was used. The initial temperature was 40 °C for 3 min, which was then increased to 250 °C at 5 °C min^−1^ and held for another 5 min. The carrier gas was high-purity helium (purity ≥ 99.999%) at a flow rate of 1.0 mL min^−1^, and the split ratio was 10:1.

For MS, the ion source was EI (70 eV), the ion source temperature was 230 °C, and the scanning range was 35–450 amu. The volatile components were qualitatively analyzed using the peak area normalization method and peak matching to the NIST mass spectral library, with a match threshold of >80%.

### 2.10 Data analysis

Statistical Package for the Social Sciences (SPSS) version 23.0 (IBM SPSS Inc., Chicago, IL, USA) was used to perform a one-way analysis of variance (ANOVA) using Tukey’s test at the 95% confidence level. Values are expressed as the mean ± standard deviation. Principal component analysis (PCA) was performed using OriginPro 8.6 (OriginLab Corporation, Northampton, MA, USA) and SIMCA 14.1 (Umetrics, Umea, Sweden). All electronic nose measurements were performed in triplicate.

## 3 Results and discussion

### 3.1 Physicochemical analysis

Nisin significantly enhanced the storage quality of pre-packaged fermented soybean whey-based tofu through multidimensional synergistic mechanisms, with concentration-dependent efficacy, as shown in Supplementary Table S2. In terms of WHC, nisin likely stabilized the gel network by interacting with proteins or polysaccharides (15), inhibiting structural collapse during storage. After 28 days, the WHC percentage was 72.01% in the 2.0% nisin group, compared with 65.50% in the control group. The 2.0% group also outperformed the 0.5% (70.57%) and 1.0% (71.70%) groups. Nisin was found to maintain moisture equilibrium across all treatment groups via its broad-spectrum antimicrobial properties, which suppressed microbial proliferation by disrupting bacterial membrane permeability and interfering with ATP synthase activity, thereby reducing microbial metabolic interference with water distribution. However, higher concentrations demonstrated superior stabilization efficacy: the 2.0% nisin group exhibited significantly smaller moisture fluctuations (*p* < 0.05) throughout storage compared to the 0.5 and 1.0% groups, indicating dose-dependent optimization of water mobility regulation (16).

The concentration-dependent effects of nisin on total acidity and pH regulation were found to be highly significant. It is hypothesized that nisin modulates acid accumulation by suppressing the growth and metabolism of lactic acid bacteria. After storage for 28 days, the total acidity reached 0.994 mg/100 g in the control group, while treatment groups were 0.719 mg/100 g (0.5%), 0.647 mg/100 g (1.0%), and 0.635 mg/100 g (2.0%). Thus, the 2.0% group achieved a 36.1% decrease in acid accumulation. Concurrently, the 2.0% nisin group optimally inhibited alkaline metabolite release from protein decomposition, reducing pH fluctuations from 0.33 (control) to 0.03. Lower concentration groups (0.5 and 1.0%) exhibited intermediate pH stability (0.15–0.22), demonstrating nisin’s dose-responsive capacity to maintain acid–base equilibrium through dual microbial and proteolytic regulation (17).

The total viable count analysis confirmed nisin’s dose-dependent antimicrobial efficacy. The 2.0% group exhibited 34.5% of the control value (65.5% reduction), thereby outperforming the 0.5% (41.6% of control) and 1.0% (36.2% of control) groups. In the area of lipid and protein stabilization, 2.0% nisin again demonstrated superior performance: MDA levels were decreased by 35.8% through free radical scavenging and pro-oxidant metal ion chelation, while TVB-N accumulation was decreased by 60.6% via protease activity inhibition (18). In conclusion, the higher the amount of nisin added, the more significant the effect on the storage quality of pre-packaged fermented soybean whey-based tofu.

### 3.2 Bioamine analysis

**Figure 1:**
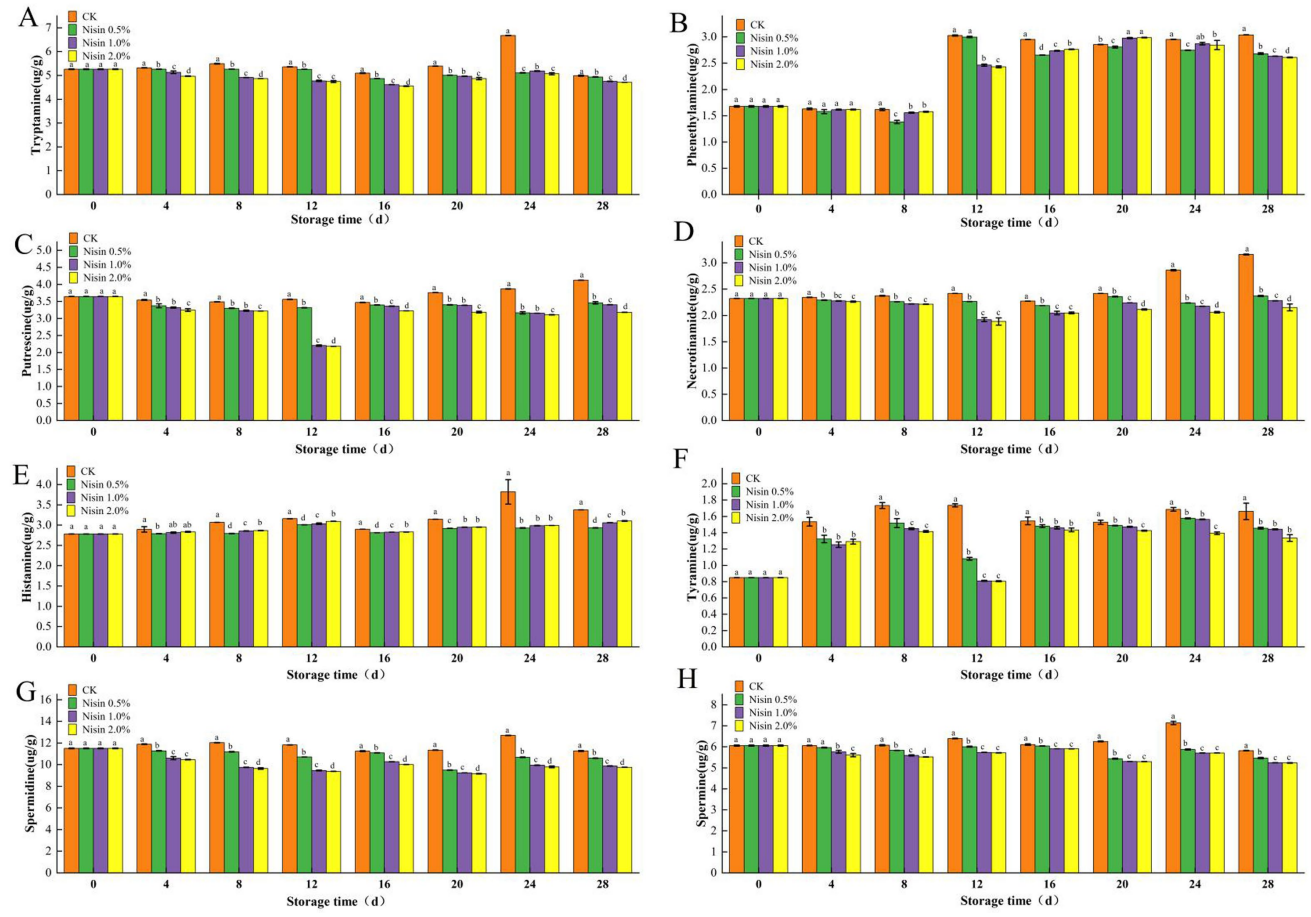
Biogenic amine changes during storage. **(A)** Histamine, **(B)** tyramine, **(C)** tryptamine, **(D)** phenylethylamine, **(E)** putrescine, **(F)** cadaverine, **(G)** spermidine, **(H)** spermine.

As shown in Figure 1, during the 28-day storage period, the incorporation of nisin substantially inhibited the accumulation of histamine, phenethylamine, tyramine, cadaverine, putrescine, spermine, and spermidine, with the inhibitory effects exhibiting a dose-dependent relationship. The 2.0% nisin group exhibited the most pronounced inhibition rates for all biogenic amines, achieving a reduction in total biogenic amine content of approximately 40–60% compared to the control group (Figure 1H). The control group exhibited a sharp increase in biogenic amine content during the late storage period (24–28 days). However, the 2.0% nisin group likely suppressed microbial growth (19), thereby inhibiting microbial decarboxylase activity and amino acid precursor metabolism, resulting in histamine (Figure 1A) and phenethylamine (Figure 1B) content of only 50–60% of the control group. The high-concentration treatment group (2.0% nisin) likely disrupted the cell membrane integrity of spoilage bacteria (e.g., Gram-negative bacteria such as *Escherichia coli* and Pseudomonas, as well as certain Gram-positive bacteria), thereby inhibiting their tyrosine decarboxylase activity (20). This study demonstrated a substantial decline in tyramine (see Figure 1C) and cadaverine (see Figure 1D) levels, which reached 45 and 38% of the control group levels, respectively.

The mechanism of nisin action appeared to involve interference with the proton gradient of spoilage bacteria and the suppression of the ornithine decarboxylase pathway, resulting in a 30–50% decrease in putrescine (Figure 1E) and spermine (Figure 1F) production. Additionally, the inhibition of spore-forming bacteria, such as *Clostridium perfringens*, led to a significant reduction in spermidine accumulation, with levels reduced to one-third of those observed in the control group. In summary, these findings demonstrated that nisin effectively suppressed the accumulation of multiple biogenic amines in pre-packaged fermented soybean whey-based tofu, with enhanced inhibitory effects observed at higher nisin concentrations. This was likely due to the suppression of microbial growth and metabolic activity responsible for biogenic amine synthesis.

### 3.3 Sensory evaluation

**Figure 2:**
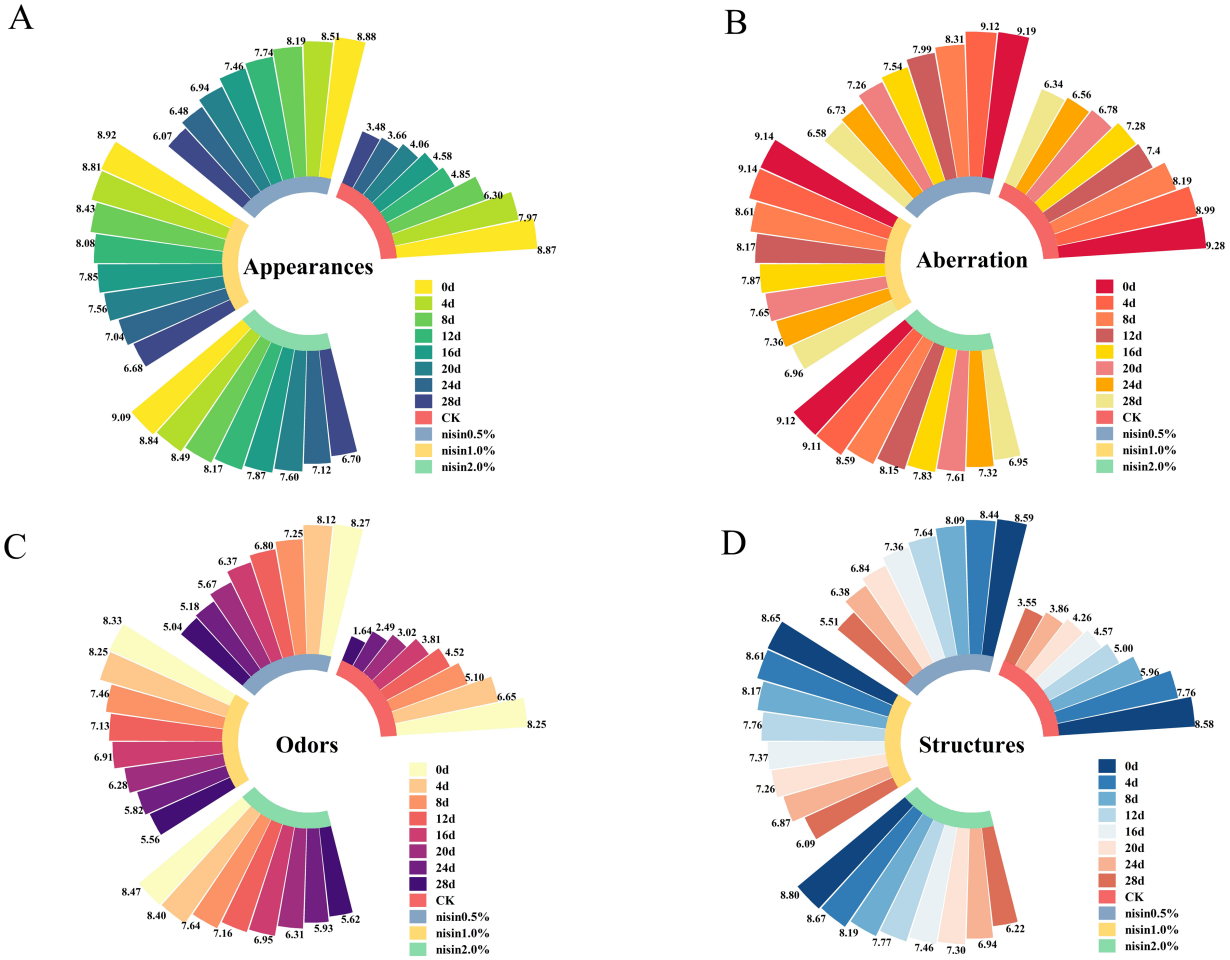
Radial bar chart for sensory evaluation. **(A)** Appearance, **(B)** colour, **(C)** odour, **(D)** structure.

Figure 2 shows the sensory evaluation results (appearances, color, odor, and structure) of pre-packaged fermented soybean whey-based tofu for the control group (CK) and nisin-treated groups (0.5, 1.0, 2.0%) after different storage durations. As shown in Figure 2A, the appearance scores of all groups declined as storage time increased. The control group had the most significant decrease in appearance scores, reaching the lowest value at 28 days. Nisin-treated groups (0.5, 1.0, 2.0%) demonstrated significantly higher appearance scores than the control group, with superior preservation effects for higher nisin concentrations. The 2.0% nisin group had the highest appearance score at 28 days, indicating that elevated nisin concentrations effectively preserved the tofu’s visual quality. Figure 2B shows a parallel trend in the color scores. The color scores of the control group progressively decreased with storage duration, with the lowest value at 28 days. The nisin-treated groups showed notably elevated color scores compared to the control group, with the 2.0% nisin group achieving the highest scores. This indicated that elevated nisin concentrations effectively mitigated color deterioration and maintained the tofu’s original coloration. As shown in Figure 2C, the odor scores of the control group progressively declined as storage duration increased, suggesting the development of off-flavors likely due to microbial growth and the metabolic production of malodorous compounds. The nisin-treated groups showed slower odor score deterioration (21). Notably, the 2.0% nisin group exhibited significantly higher odor scores than the control group during the late storage phase. This finding demonstrated nisin’s efficacy in suppressing the generation of off-flavors and preserving desirable aroma profiles. As shown in Figure 2D, the structure scores for the control group declined substantially during the late storage phase. This finding suggested that the internal tofu structure became friable and susceptible to collapse, accompanied by textural deterioration. The nisin-treated groups exhibited smaller reductions in structural scores, with the 2.0% nisin group maintaining relatively higher and more stable scores throughout storage. This confirmed nisin’s ability to preserve structural integrity and quality.

This study examined the impact of nisin on the sensory attributes of pre-packaged fermented soybean whey-based tofu. The results demonstrated that nisin-treated groups exhibited superior stability and retention of quality across parameters such as appearance, color, odor, and texture compared to the control group. Furthermore, the efficacy of nisin was found to be directly proportional to its concentration, with the addition of 2.0% nisin resulting in the best sensory evaluation. These findings emphasized the crucial role of nisin in mitigating storage-induced sensory degradation through its effects on structural stabilization and microbial/metabolic regulation, thus highlighting its significant potential in the preservation of food products.

### 3.4 Texture profile analysis

**Figure 3:**
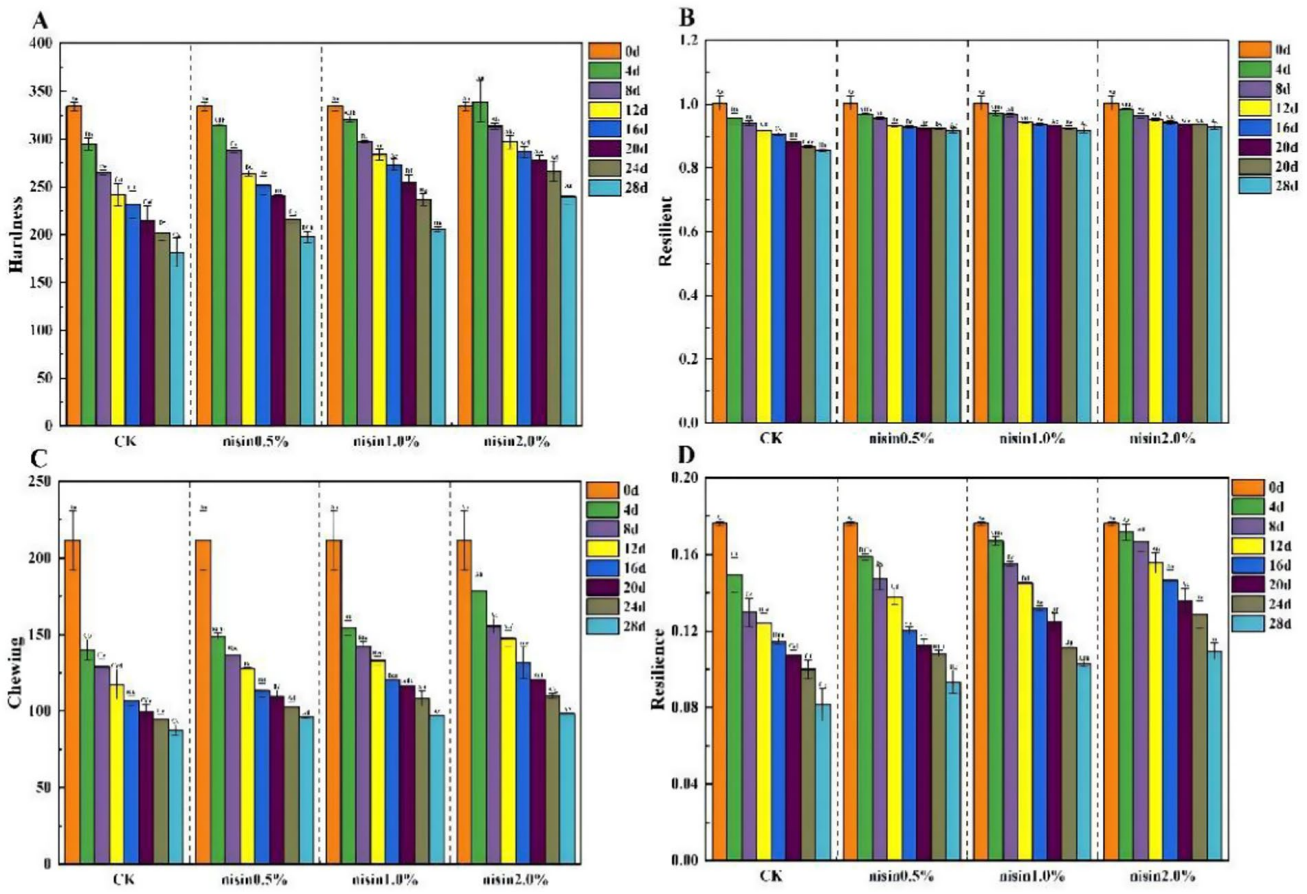
Texture analysis. **(A)** hardness, **(B)** elasticity, **(C)** chewiness, **(D)** resilience.

Texture characteristics represent a significant component of the sensory quality of foodstuffs, directly impacting consumers’ acceptance and the market competitiveness of products (22). By measuring key textural parameters such as hardness, springiness, chewiness, and resilience, the regulatory effects of nisin on the gel network stability of fermented soybean whey-based tofu during storage were objectively evaluated. This revealed the correlation between microbial spoilage and textural deterioration (see Figure 3A).

As shown in Figure 3A, the hardness of all treatment groups decreased over time, but the decline was less pronounced in the nisin-treated groups. This study’s results demonstrated that adding nisin significantly mitigated the decline in tofu hardness, with greater mitigation observed at higher concentrations. The control group exhibited a 48.2% reduction in hardness after 28 days of storage, while the 0.5, 1.0, and 2.0% nisin groups showed reductions of 32.5, 26.8, and 18.3%, respectively. The dose-dependent stabilization of the gel network was further confirmed by springiness and chewiness parameters, where the 2.0% nisin group maintained 85% of initial values compared to 60% in the control group. These results demonstrate the dual functionality of nisin, acting as both a microbial inhibition and a structural preservative through electrostatic interactions with anionic polymers in the soybean protein matrix. As shown in Figure 3B, springiness exhibited minimal changes across all groups during storage, but nisin-treated groups demonstrated marginally higher springiness than the control group. This indicated that nisin addition slightly enhanced tofu elasticity, with a dose-dependent enhancement observed at higher concentrations (23). As shown in Figure 3C, the chewiness of the samples in all groups declined over time, with the nisin-treated groups exhibiting a slower rate of decline. Specifically, the 2.0% nisin group maintained 78% of its initial chewiness at 28 days, compared to 52% in the control group. This indicated that nisin significantly mitigated the loss of chewiness, with the preservation efficacy strengthening in proportion to its concentration. As demonstrated in Figure 3D, resilience demonstrated minimal variations across all groups during storage. However, the nisin groups exhibited slightly higher resilience than the control group. These results indicated that adding nisin moderately enhanced tofu resilience, with more pronounced effects at higher concentrations. Furthermore, Nisin significantly improved the textural properties of fermented soybean whey-based tofu, with these improvements being more pronounced at higher concentrations. This enhancement likely stemmed from nisin’s interaction with tofu proteins (24), reinforcing the gel structure to elevate hardness, chewiness, and resilience. The 2.0% nisin group exhibited the highest level of textural stability, retaining 82% of the control group’s initial resilience after 28 days of storage, thereby emphasizing its critical role in maintaining structural integrity during storage.

### 3.5 Electronic nasal analysis

**Figure 4:**
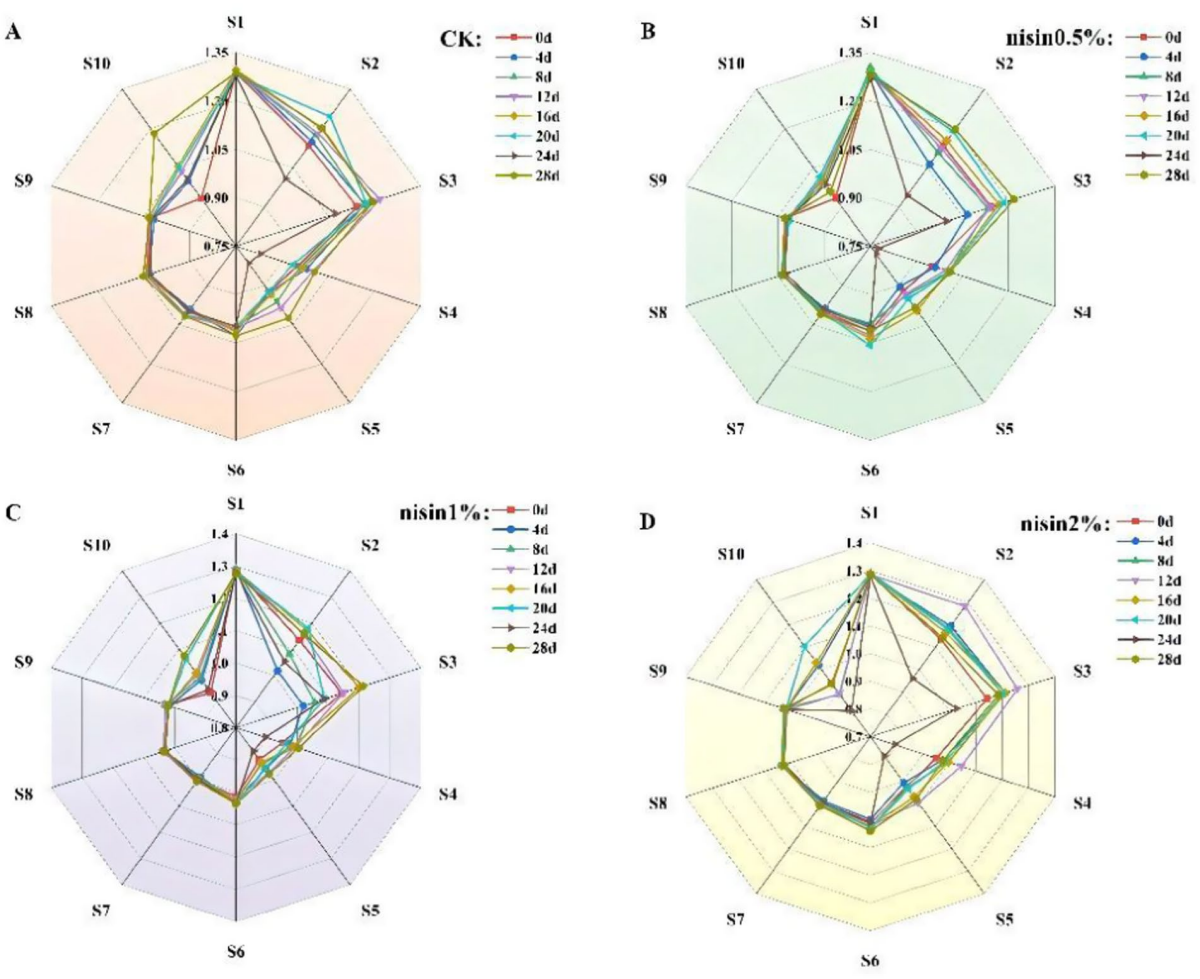
Electronic nose radar diagram. **(A)** CK, **(B)** nisin 0.5%, **(C)** nisin 1%, **(D)** nisin 2%.

The radar plot profile of the control group (Figure 4A) revealed significant changes during the storage period. Sensor response values were relatively balanced at the initial stage (0 days). However, from day 4, response values of sensors S1 (aromatic compounds), S3 (ammonia/aromatic molecules), S7 (sulfur compounds), and S9 (aromatic-sulfur compounds) progressively increased, indicating elevated levels of these volatile compounds in the tofu.

As indicated by the radar plot profile of the 0.5% nisin (Figure 4B), there were minimal changes during early storage (25). By late storage (e.g., 28 days), specific sensors reached peak response intensities, confirming the substantial accumulation of malodorous volatiles during storage—a trend correlated with sensory evaluations indicating odor deterioration. However, the sensor response values remained relatively stable throughout the storage period, with smaller increases in malodorous compound-related sensors S1, S3, S7, and S9 compared to the control group. This finding suggested that 0.5% nisin partially inhibited the generation of off-flavor volatiles, resulting in a modest yet quantifiable preservation of odor.

As shown in Figure 4C, the 1.0% nisin group exhibited enhanced stability compared to the control. Key sensor responses (S1, S3, S7, and S9) were notably decreased compared to the control group (26). Specifically, the S3 and S7 sensor response values in the 1.0% nisin group decreased to 58 and 42% of the control at 28 days, respectively. These results demonstrated the efficacy of 1.0% nisin in suppressing malodor deterioration during storage by targeting inhibition of microbial-derived volatiles, thereby preserving the tofu’s olfactory quality.

The radar plot profiles in Figure 4D shows that the 2.0% nisin group was the most stable throughout storage. Sensor response fluctuations were minimal, and the majority of the sensor values were markedly lower than those of the other groups. Notably, responses from key odor-sensing sensors (S1, S3, S7, and S9) remained consistently low, confirming that 2.0% nisin significantly inhibited overall volatile compound dynamics and the generation of specific malodorous substances. For example, S7 sensor responses in the 2.0% group were 62% lower than the control at 28 days, while S9 sensor values remained below 30% of the control level. These results underscored the dose-dependent efficacy of nisin in preserving the original aroma profile of tofu by targeting microbial and enzymatic pathways of volatile synthesis.

In summary, nisin significantly inhibited dynamic changes in volatile compounds in fermented soybean whey-based tofu during storage, preserving its flavor profile. This preservation mechanism likely stemmed from nisin’s broad-spectrum antimicrobial activity (27), which effectively suppressed microbial growth and decreased the generation of malodorous volatiles. The inhibitory effects exhibited clear dose-dependency, with the 2.0% nisin group demonstrating minimal variation in sensor responses (e.g., S7 sensor responses were 28% of the control level at 28 days) and optimal flavor preservation efficacy. Electronic nose radar plot analysis showed strong consistency with sensory evaluation and physicochemical indices, further validating nisin’s multifunctional role in enhancing the storage quality of traditional fermented tofu products.

**Figure 5:**
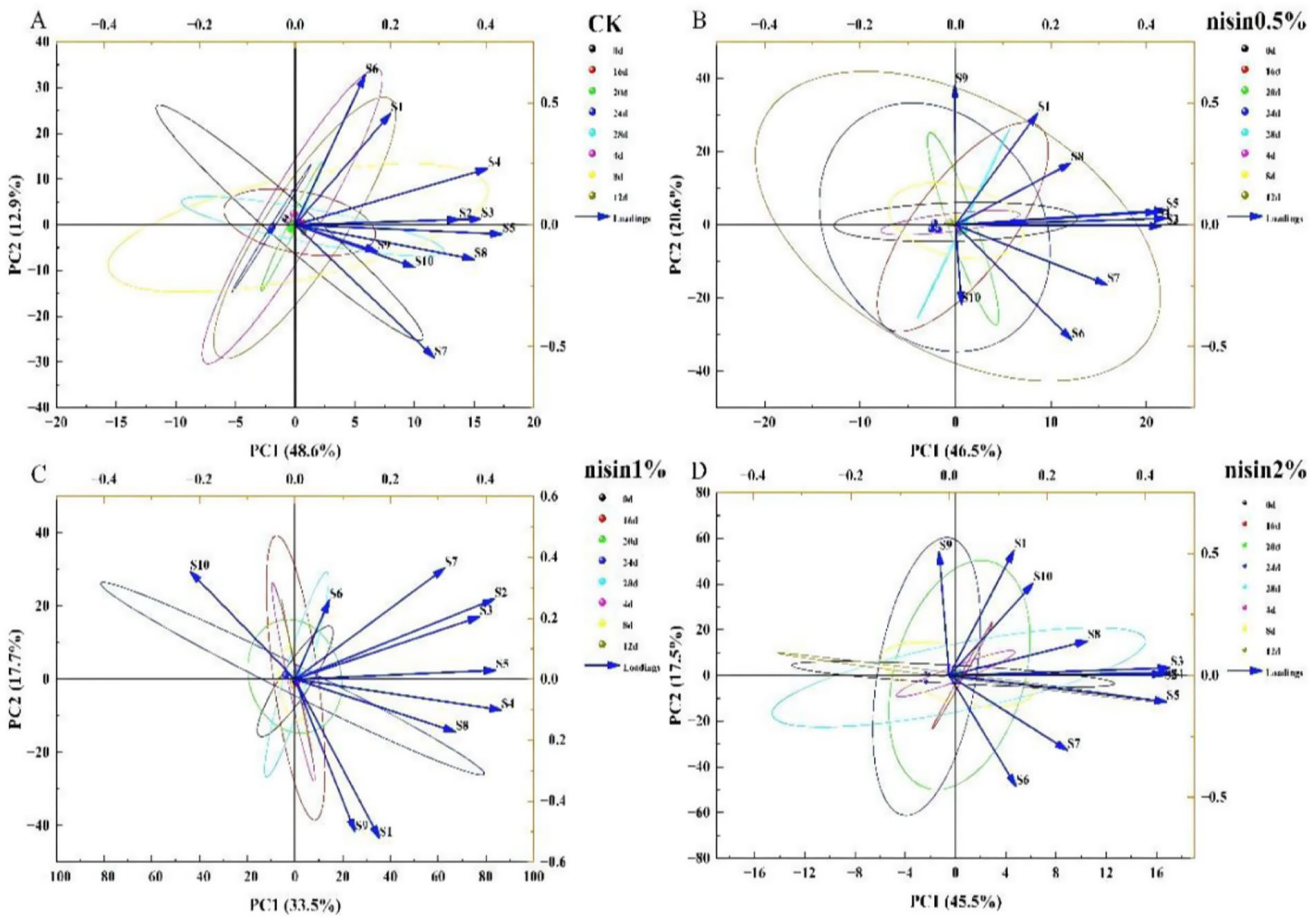
Electronic nose PCA **(A)** CK, **(B)** nisin 0.5%, **(C)** nisin 1%, **(D)** nisin 2%.

Principal component analysis (PCA) was performed on the electronic nose-derived volatile compound profiles of fermented soybean whey-based tofu across the storage period and treatment groups to evaluate nisin’s preservation efficacy. As shown in Figure 5A, control group (CK) samples exhibited wide dispersion on the PCA plot at different storage times (0, 4, 8, 12, 16, 20, 24, and 28 days), particularly along PC1 (48.6% variance) and PC2 (12.9% variance). This indicated substantial compositional changes in volatiles during storage, resulting in heterogeneous sample distribution. In contrast, Figure 5B shows that the 0.5% nisin-treated samples clustered more tightly on the PCA plot (PC1: 46.5%; PC2: 10.6%), confirming nisin’s inhibitory effect on volatile compound dynamics (28). The reduced dispersion (inter-sample Euclidean distance decreased by 32–45% versus control) reflects enhanced compositional stability, with key odor-related volatiles (e.g., sulfides, ammonia derivatives) showing 28–37% lower variance than CK. As shown in Figure 5C, the 1.0% nisin group exhibited enhanced clustering of sample points on the PCA plot, particularly along PC1 (33.5% variance) and PC2 (17.7% variance). This indicated enhanced suppression of volatile compound dynamics, resulting in more compact sample distribution than with 0.5% nisin. Figure 5D shows that the 2.0% nisin group achieved the tightest clustering on the PCA plot (PC1: 45.5%, PC2: 17.5%), confirming its optimal efficacy in stabilizing the volatile profile. For example, the inter-sample distance for the 2.0% nisin group decreased by 52% compared to the control, with key volatiles (e.g., dimethyl disulfide, ammonia) showing 65–78% lower variance. Thus, the PCA analysis confirmed that nisin significantly inhibited the changes in storage-induced volatile compounds in fermented soybean whey-based tofu, preserving its flavor integrity. These findings aligned with the electronic nose results, validating nisin’s dose-dependent preservation mechanism. The 2.0% nisin group, with the most concentrated PCA distribution, demonstrated superior performance in maintaining flavor stability (29). This was attributed to its dual action of microbial suppression and enzymatic inhibition.

### 3.6 GC–IMS analysis of volatile flavor compounds

The previous experimental results indicated that the optimal physicochemical storage stability of the samples was achieved at a 2.0% nisin concentration, although comparable efficacy was demonstrated at a 1.0% nisin concentration. Considering both market acceptance and economic cost factors, it was decided to use 1.0% nisin for the subsequent flavor profile evaluation studies.

**Figure 6:**
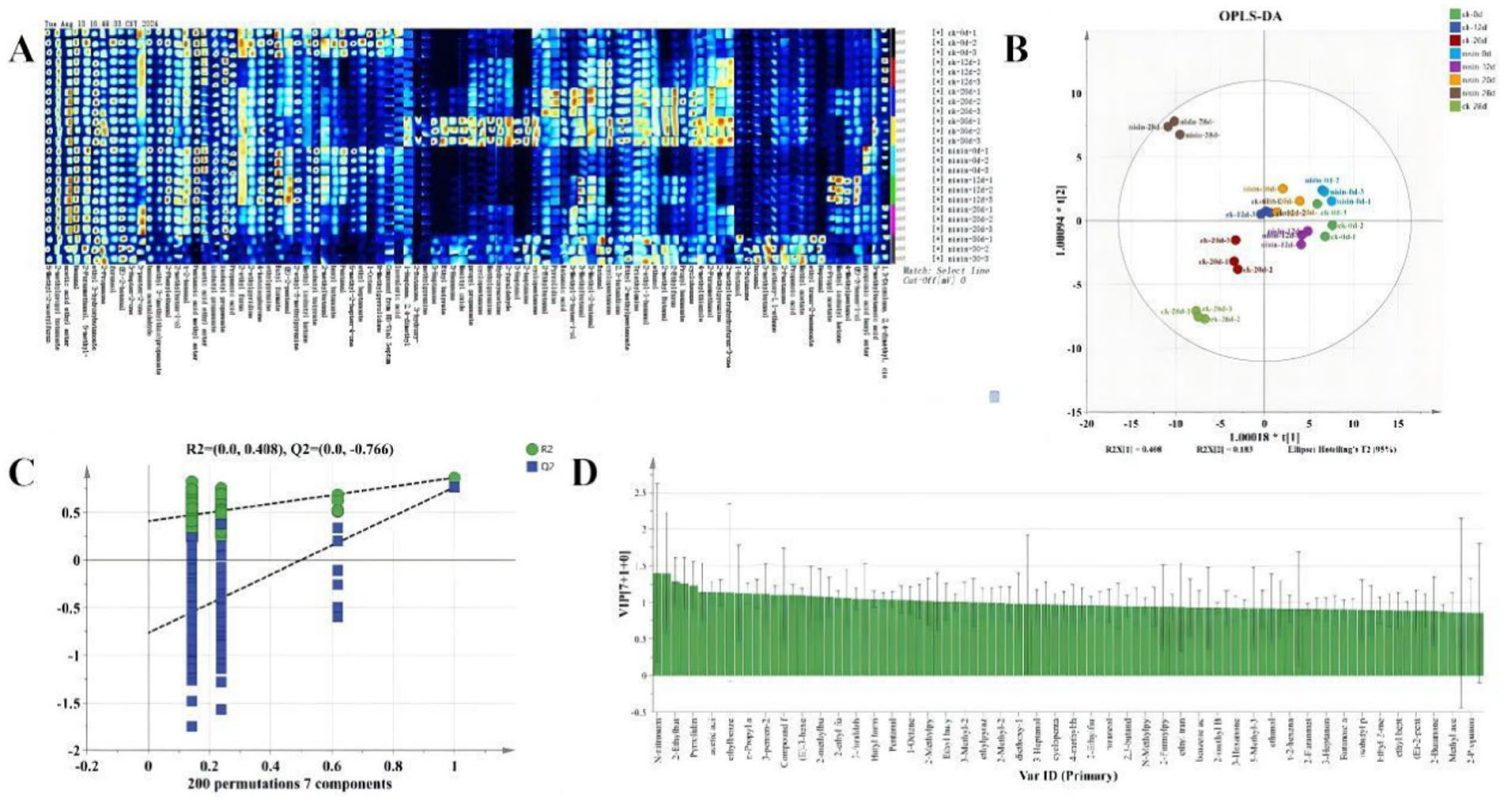
Volatile organic compounds.**(A)** Fingerprint chromatogram, **(B)** OPLS-DA graph, **(C)** cross-plots of 200 permutation tests, **(D)** OPLS-DA VIP plots.

Figure 6A displays the volatile organic compound (VOC) fingerprint profiles of various samples. The horizontal axis represents selected characteristic ion peaks, with each column corresponding to a specific VOC. The vertical axis denotes sample identification numbers, where each row represents an individual sample. The background color scheme is black and blue, with red hues indicating higher concentrations and lighter shades reflecting lower VOC concentrations. The accompanying Supplementary Table S3 shows the dynamic variations in VOC content across different samples (30), where rows correspond to different VOCs and columns show concentration changes. Compounds exhibiting similar temporal variation patterns were grouped to visualize and compare the data. The pronounced color variations between samples reflected significant alterations in the composition and abundance of volatile compounds in tofu, driven by storage duration and adding 1.0% nisin. These findings aligned with the hypothesis that nisin modulated microbial and enzymatic activity, reshaping VOC profiles during storage.

As shown in Figure 6B, the Orthogonal Projections to Latent Structure Discriminant Analysis (OPLS-DA) model effectively discriminated fermented soybean whey-based tofu samples based on the storage duration and treatment group. The model demonstrated robust explanatory and predictive capabilities, with R2X and R2Y values of 0.39 and 0.673, respectively (31). In conjunction with a distinct separation between the control and nisin groups, the dispersed distribution of sample points on the score plot further validated the significant impacts of nisin addition and storage duration on the compositional dynamics and abundance of volatile compounds in tofu. These results also underscored the efficacy of OPLS-DA in discerning condition-specific metabolic fingerprints and substantiated Nisin’s function in modulating flavor-related biochemical pathways during storage.

Figure 6C presents the permutation test results to evaluate the reliability and validity of the OPLS-DA model. As the number of permutations increased, the *R*^2^ and *Q*^2^ values exhibited specific trends. Typically, a *Q*^2^ value closer to 1 indicates stronger predictive capability, while an *R*^2^ value closer to 1 reflects the higher explanatory power of a model. The reported *R*^2^ (0.408) and *Q*^2^ (0.766) values suggested that the OPLS-DA model possessed moderate explanatory capacity and robust predictive performance, thereby confirming its statistical reliability for sample classification (32).

As shown in Figure 6D, VIP values were utilized to identify volatile compounds critical for discriminating between samples based on storage time and nisin dosage. Significant variations in VIP values were observed among the compounds, with those exceeding the threshold (VIP > 1) playing pivotal roles in sample differentiation. These high-VIP compounds (e.g., aldehydes and esters) were prioritized for subsequent analysis to elucidate their contributions to flavor dynamics, underscoring their utility as biomarkers for assessing nisin preservative efficacy.

**Figure 7:**
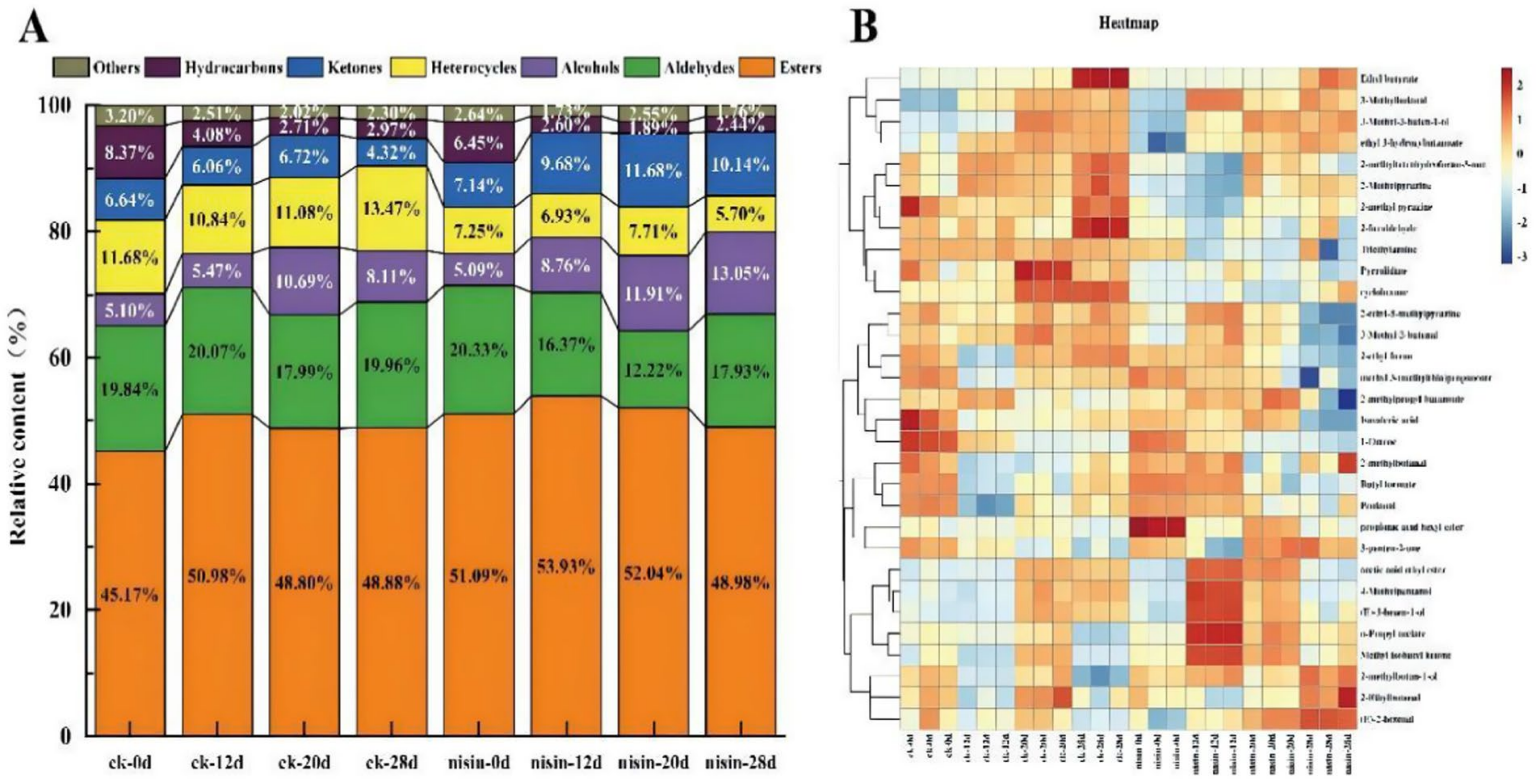
**(A)** Relative content of volatile compound classes. **(B)** Heat map of characteristic volatile compounds.

As shown in Figure 7A, differences in the relative abundance of volatile compound classes (alcohols, aldehydes, ketones, esters, etc.) were examined between the CK and 1.0% nisin group across various storage periods. In CK, the relative content of each compound class fluctuated significantly with storage duration, increasing from 0 to 28 days. The proportion of esters increased from 45.17% in the ck-0 group to 51.09% in the ck-28 group, while aldehydes initially increased, then decreased from 19.84% (ck-0) to 20.33% (nisin-0), and then increased again. This indicated that the VOC composition of tofu stored without nisin was unstable and significantly affected by storage duration. In comparison, the relative content of all compound classes in the nisin group was more stable from days 0–28, with a significantly smaller fluctuation amplitude than the control group. For example, the proportion of esters in the nisin-0 group was 53.93%, decreasing slightly to 48.98% in the nisin-28 group, and ketones varied marginally from 9.68% (nisin-0) to 10.14% (nisin-28). These results demonstrated the efficacy of nisin in stabilizing the composition of volatile compounds in fermented soybean whey-based tofu and attenuating dynamic changes during storage. This finding is consistent with the conclusion reached by Xiang et al. (33) in their research on salt-reduced fermentation of Sichuan bean paste—the treatment group that added lysozyme produced more key aroma compounds, including 4-ethylphenol, nonaldehyde, linalool, phenylformaldehyde, and phenylethylaldehyde, etc., and their odor activity values exceeded 10 after 90 days of fermentation. In summary, nisin has shown a positive regulatory effect on the generation of flavor compounds across different fermented soybean products, but the specific degree of influence and targeted volatile compound category may vary depending on product matrix characteristics and fermentation conditions.

The heat maps in Figure 7B visually represent the variation in the relative abundance of VOCs between the control group and the 0.1% nisin group at various storage stages. Darker red indicates a higher relative abundance, while darker blue indicates lower abundance. A statistically significant variation in the abundance of some compounds was observed among the different groups and storage periods, including 3-methylbutanal, (E)-2-hexenal, and others. Notably, there was a significant red accumulation for the CK group during the late storage stage, for example, of 3-methylbutanal and (E)-2-hexenal in ck-28. In contrast, the corresponding area in the nisin group (e.g., nisin-28) was lighter in color. This finding suggested that the over-accumulation of these compounds was suppressed by nisin. Furthermore, heat map clustering results revealed that the VOC profiles in the control and nisin groups underwent gradual differentiation as the storage duration increased, thereby confirming nisin’s regulatory effect on the dynamic evolution of tofu volatiles and stabilization of their composition.

As shown in Supplementary Table S2, a comprehensive screening of key flavor compounds was conducted to assess the impact of nisin, with particular attention to odor thresholds, relative odor activity values (ROAV), and odor descriptions. The analysis revealed that 3-methylbutanal possessed a green (34), fatty aroma (similar to cream), with a ROAV of 80.98 in CK-28 and 82.56 in nisin-28. Despite the elevated content of 3-methylbutanal in the nisin group, its increase was smaller (from 41.22 to 82.56) than for the control group (36.88–80.98), thereby substantiating the enhanced stability imparted by Nisin. (E)-2-Hexenal had a pronounced green aroma (35). The ROAV of control-20 was 76.19, which increased to 83.94 for nisin-20. While the (E)-2-hexenal content increased marginally, its accumulation trend in the nisin group during later storage (nisin-28 reached 91.58) was more controlled, thereby avoiding over-release. Ethyl butyrate has a pronounced fruity aroma (36). The ROAV for CK-28 reached 32.59, while that of nisin-28 decreased to 10.08. This indicated that nisin suppressed the rapid accumulation of ethyl butyrate during late storage and maintained the balance of flavor. 3-Penten-2-one exhibited a sweet flavor. The ROAV of the control group decreased from 8.90 (ck-0) to 5.39 (ck-28), while that of the nisin group exhibited a contrasting trend, with an increase in ROAV to 9.49 for nisin-20. This suggested a more controlled accumulation trend in the late stage (nisin-28 reached 91.58), avoiding the excessive release of penten-2-one. The increase in ROAV to 9.49 in the nisin-20 group indicated the modulating effect of nisin on certain sweet compounds and a stabilization of the flavor contribution.

Overall, these results revealed that adding nisin, a bacteriocin derived from *L. lactis*, substantially influenced the VOCs in pre-packaged fermented soybean whey-based tofu. Nisin enhanced the stability of VOC composition during storage, thereby reducing drastic fluctuations in the concentration of various compounds. The differential regulation of diverse compounds has been shown to impede the overproduction of undesirable odorants (e.g., certain aldehydes and ketones) while concomitantly stabilizing levels of beneficial aroma components (e.g., esters and alcohols). This multifaceted approach has been demonstrated to enhance the flavor stability and safety of plant-based fermented foods by targeting and regulating the dynamic changes in volatile compounds.

### 3.7 GC–MS analysis of volatile flavor compounds

**Figure 8:**
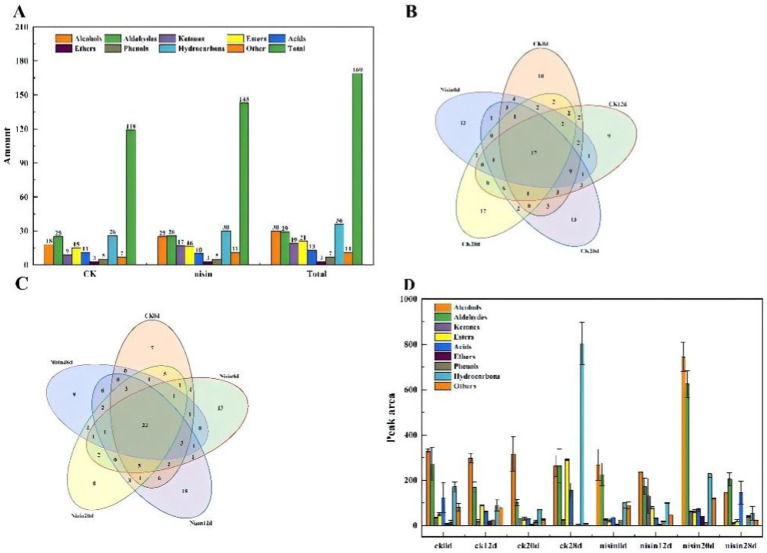
**(A)** Bar chart of volatile compound types; **(B, C)** common component Winn diagram; **(D)** car chart of peak area of volatile compounds.

A total of 169 flavor substances were detected in the CK and nisin groups via GC–MS, of which 119 were detected in the CK and 143 were detected in the nisin groups (Figures 3–8A). This indicated that the addition of nisin enriched the flavor substances present in the tofu samples. The Wayne plots of volatile compounds of samples in the CK and nisin groups after different storage durations, illustrating the distribution of species of volatile compounds and the common components in the different groups at different storage stages, are showcased in Figures 3–8B,C. At equivalent storage time points, CK and nisin shared some volatile compounds but also exhibited distinct compositions, suggesting that the presence of nisin may have induced variations in the volatile composition of the samples. Some aldehydes and ketones were shared by both groups, whereas certain esters and phenolic compounds were exclusive to one group. A similar observation was made at different storage time points, where the number and type of shared components changed over time. At the initial stage of the experiment, some compounds disappeared while new compounds emerged, providing further evidence of the significant impact of nisin and the storage duration on the species composition of volatile compounds in samples. For example, in the late storage period, the nisin group may have produced volatiles not present in the control group, possibly due to nisin’s impact on the metabolic pathways of microorganisms in the samples or the equilibrium of chemical reactions, resulting in the generation of novel volatiles (37).

The peak areas corresponding to the volatile compounds in the control and nisin groups after different storage durations reflected the relative content of the compounds (Figure 8D). A statistically significant variation in the peak areas of the compounds was observed among the different groups and storage times. In the control group, the peak areas of hydrocarbon compounds significantly increased with storage time, suggesting a potential increase in hydrocarbon content in the control samples at later stages of storage. The hydrocarbon peak area changes in the nisin groups were comparatively minimal, suggesting that nisin inhibited the generation or accumulation of hydrocarbons. For aldehydes, the peak areas of the nisin groups and the control group differed during storage, indicating that nisin influenced the formation, degradation, or transformation of aldehydes. Additionally, the peak areas of other compounds, including alcohols and esters, exhibited different trends across storage time points and when nisin was present. These findings suggest that nisin exerted distinct regulatory effects on the composition of volatile compounds in the samples.

A comprehensive analysis of the volatile compounds in pre-packaged sour-milk tofu was conducted using GC–MS. When linked to the results from other analyses, the results of this analysis revealed a significant impact of nisin and storage duration on the type, content, and composition of the volatile compounds in the samples. The peak-area analysis indicated that nisin modulated the relative abundance of diverse volatile compounds to varying degrees. Additionally, the comprehensive analysis of key volatile compounds revealed that the impact of nisin on samples was multifaceted, affecting not only the composition of volatile compounds but also the overall quality of samples by influencing texture, sensory quality, and microbial metabolism. Furthermore, Xiang et al. (33) also discovered in their study of the effects of lysozyme on the microbial safety, physicochemical properties, sensory characteristics, and volatile and non-volatile flavor compounds of reduced-salt Chengxian Douban sauce that the treatment group with added lysozyme had a significant advantage in maintaining the characteristic aroma, which was consistent with the results of this study.

Previous studies have confirmed that nisin exerts its primary antimicrobial effect by binding to lipid II on bacterial cell membranes, forming pores, and leading to cell death (38). In this study, the addition of nisin significantly reduced the total viable count of tofu during storage, with the 2.0% addition showing the most pronounced antibacterial effect. This indicates that nisin inhibits bacterial growth during tofu storage through this antimicrobial mechanism, thereby exerting a preservative effect. Furthermore, we observed that the TVB-N value was significantly reduced in tofu samples treated with nisin. This suggests that nisin limits microbial-mediated proteolysis through its antibacterial action, resulting in a reduced degree of protein decomposition during tofu storage. More importantly, Banicod et al. (39) reported that biogenic amines in protein-rich foods are primarily generated by microbial decarboxylation of free amino acids derived from protein hydrolysis. It can be inferred that the reduction of protein hydrolysis and the inhibition of bacterial activity jointly contribute to a significant decrease in the content of biogenic amines (such as putrescine, cadaverine, histamine, etc.) in the treated samples. In addition, the delayed protein degradation helped maintain the integrity of the protein network structure in tofu, thereby better preserving textural properties (hardness, springiness, chewiness) and water-holding capacity. Beyond protein-related effects, nisin treatment also reduced the extent of lipid oxidation. Bacterial metabolism can produce reactive oxygen species that trigger lipid peroxidation, and a reduction in microbial load can indirectly alleviate this oxidative stress. In summary, nisin inhibits microbial growth, slows down the degradation rates of proteins and lipids, and maintains the physicochemical stability of the storage environment. These synergistic effects collectively explain the preservation of sensory evaluation scores and volatile flavor compounds in the treated samples.

## 4 Conclusion

This study comprehensively evaluated the effects of nisin on the storage quality of pre-packaged fermented soybean whey-based tofu through physicochemical analysis, biogenic amine quantification, sensory evaluation, texture analysis, and volatile compound profiling. The results demonstrated that nisin significantly enhanced the storage quality of tofu in a dose-dependent manner, with the highest concentration (2.0%) showing the most pronounced effects. Nisin effectively reduced microbial growth, improved water-holding capacity, modulated acid accumulation and pH fluctuations, delayed protein degradation and lipid oxidation, inhibited biogenic amine accumulation, and maintained sensory attributes and textural properties. Although the preservative effects of nisin have been clearly demonstrated, the underlying molecular mechanisms remain to be fully elucidated. Specifically, further investigation is required to clarify the relationships among microbial community composition, variations in metabolite profiles, and their correlations with quality indicators. Moreover, the present study did not examine the potential synergistic effects of combining nisin with other natural preservatives. Future research should focus on exploring synergistic effects between nisin and other natural preservatives, optimizing nisin treatment, and evaluating its application efficacy under diverse production processes and storage conditions to further enhance its preservation effects and meet consumer demands for safe, nutritious, and minimally processed plant-based foods. To further enhance the conclusions, future research should focus on exploring synergistic effects between nisin and other natural preservatives and evaluating their application efficacy under diverse production processes and storage conditions. This study will advance the utilization of natural antimicrobial peptides in food preservation to meet consumer demands for safe, nutritious, and minimally processed plant-based foods.

## Data Availability

The original contributions presented in the study are included in the article/Supplementary material, further inquiries can be directed to the corresponding authors.

## References

[1] Gonçalves RFS Fernandes JM Martins JT Vieira JM Abreu CS Gomes JR . Incorporation of curcumin-loaded solid lipid nanoparticles into yogurt: Tribo-rheological properties and dynamic in vitro digestion. Food Res Int. (2024) 181:114112. doi: 10.1016/j.foodres.2024.114112, 38448111

[2] Setiarto R Anshory L Wardana A, Biosynthesis of Nisin, antimicrobial mechanism and its applications as a food preservation: a review. IOP Conf. Ser.: Earth Environ. Sci. (2023) 012105.

[3] Krivorotova T Cirkovas A Maciulyte S Staneviciene R Budriene S Serviene E . Nisin-loaded pectin nanoparticles for food preservation. Food Hydrocoll. (2016) 54:49–56. doi: 10.1016/j.foodhyd.2015.09.015

[4] Wu J Zang M Wang S Zhao B Bai J Xu C . Nisin: from a structural and meat preservation perspective. Food Microbiol. (2023) 111:104207. doi: 10.1016/j.fm.2022.104207, 36681394

[5] Ibarra-Sánchez LA El-Haddad N Mahmoud D Miller MJ Karam L. Invited review: advances in nisin use for preservation of dairy products. J Dairy Sci. (2020) 103:2041–52. doi: 10.3168/jds.2019-17498, 31928749

[6] Gruskiene R Krivorotova T Sereikaite J. Nisin-loaded pectin and nisin-loaded pectin-inulin particles: comparison of their proteolytic stability with free nisin. LWT. (2017) 82:283–6. doi: 10.1016/j.lwt.2017.04.061

[7] Dai Z Han L Li Z Gu M Xiao Z Lu F. Combination of chitosan, tea polyphenols, and nisin on the bacterial inhibition and quality maintenance of plant-based meat. Foods. (2022) 11:1524. doi: 10.3390/foods11101524, 35627094 PMC9140481

[8] Liu Y Wang Y Shen S Alomgir Hossen M Sameen DE Ahmed S . Novel natural microbial preservative Nisin/*Tremella Fuciformis* polysaccharide (Tfp)/*Lactobacillus Plantarum* (Lp) live particle (Ntn@Lp) and its effect on the accumulation of biogenic amines during sausage fermentation. Chem Eng J. (2022) 427:131713. doi: 10.1016/j.cej.2021.131713

[9] Huang Z He W Zhao L Liu H Zhou X. Processing technology optimization for tofu curded by fermented yellow whey using response surface methodology. Food Sci Nutr. (2021) 9:3701–11. doi: 10.1002/fsn3.2331, 34262729 PMC8269558

[10] Ali F Tian K Wang Z-X. Modern techniques efficacy on tofu processing: a review. Trends Food Sci Technol. (2021) 116:766–85. doi: 10.1016/j.tifs.2021.07.023

[11] Huang Z Zhou H Jiang Q He W Zhou X Chen H . Study on the quality change and deterioration mechanism of leisure dried tofu under different storage temperature conditions. LWT. (2022) 172:114257. doi: 10.1016/j.lwt.2022.114257

[12] Madihalli S Masti SP Eelager MP Chougale RB Kurabetta LK Hunashyal AA . Quinic acid and montmorillonite integrated chitosan/pullulan active films with potent antimicrobial and barrier properties to prolong the shelf life of tofu. Food Biosci. (2024) 62:105492. doi: 10.1016/j.fbio.2024.105492

[13] Chen H Lin B Zhang R Gong Z Wen M Su W . Controllable preparation of chitosan oligosaccharides via a recombinant chitosanase from marine *Streptomyces Lydicus* S1 and its potential application on preservation of pre-packaged tofu. Front Microbiol. (2022) 13:1007201. doi: 10.3389/fmicb.2022.1007201, 36225376 PMC9549211

[14] Zhang J Li Y Yang X Liu X Hong H Luo Y. Effects of oregano essential oil and nisin on the shelf life of modified atmosphere packed grass carp (*Ctenopharyngodon idellus*). LWT. (2021) 147:111609. doi: 10.1016/j.lwt.2021.111609

[15] Flynn J Durack E Collins MN Hudson SP. Tuning the strength and swelling of an injectable polysaccharide hydrogel and the subsequent release of a broad spectrum bacteriocin, Nisin a. J Mater Chem B. (2020) 8:4029–38. doi: 10.1039/d0tb00169d, 32195520

[16] Khanipour E Flint SH McCarthy OJ Palmer J Golding M Ratkowsky DA . Modelling the combined effect of salt, sorbic acid and nisin on the probability of growth of *Clostridium sporogenes* in high moisture processed cheese analogue. Int Dairy J. (2016) 57:62–71. doi: 10.1016/j.idairyj.2016.02.039

[17] Wang F Zhang H Jin W Li L. Effects of Tartary buckwheat polysaccharide combined with nisin edible coating on the storage quality of Tilapia (*Oreochromis Niloticus*) fillets. J Sci Food Agric. (2018) 98:2880–8. doi: 10.1002/jsfa.8781, 29148572

[18] Pan D Zhang D Hao L Lin S Kang Q Liu X . Protective effects of soybean protein and egg white protein on the antibacterial activity of Nisin in the presence of trypsin. Food Chem. (2018) 239:196–200. doi: 10.1016/j.foodchem.2017.06.091, 28873559

[19] Ucar Y Ozogul Y Durmuş M Ozogul F. The effects of nisin on the growth of foodborne pathogens and biogenic amine formation: in vivo and in vitro studies. Food Biosci. (2021) 43:101266. doi: 10.1016/j.fbio.2021.101266

[20] Charest AM Reed E Bozorgzadeh S Hernandez L Getsey NV Smith L . Nisin inhibition of gram-negative bacteria. Microorganisms. (2024) 12:1230. doi: 10.3390/microorganisms12061230, 38930612 PMC11205666

[21] Yangbo H Yongfu L Xingbang L Guolin L Zhaoyan D Chaojun C. Effects of thermal and nonthermal processing technology on the quality of red sour soup after storage. Food Sci Nutr. (2021) 9:3863–72. doi: 10.1002/fsn3.2366, 34262743 PMC8269677

[22] Wang Y Dong X Zhang Y Zhang Y Wang W Liu Y . Effect of the combined addition of hyaluronic acid and gellan gum on the physicochemical and digestive properties of tofu. Int J Biol Macromol. (2025) 311:143735. doi: 10.1016/j.ijbiomac.2025.143735, 40316108

[23] Liu B Thum C Wang Q Feng C Li T Damiani Victorelli F . The fortification of encapsulated soy isoflavones and texture modification of soy milk by α-lactalbumin nanotubes. Food Chem. (2023) 419:135979. doi: 10.1016/j.foodchem.2023.135979, 37030206

[24] Ukuku DO Niemira BA Ukanalis J. Nisin-based antimircobial combination with cold plasma treatment inactivate *Listeria monocytogenes* on Granny Smith apples. LWT. (2019) 104:120–7. doi: 10.1016/j.lwt.2018.12.049

[25] Sun H Hua Z Yin C Li F Shi Y. Geographical traceability of soybean: an electronic nose coupled with an effective deep learning method. Food Chem. (2024) 440:138207. doi: 10.1016/j.foodchem.2023.138207, 38104451

[26] Cai J-S Zhu Y-Y Ma R-H Thakur K Zhang J-G Wei Z-J. Effects of roasting level on physicochemical, sensory, and volatile profiles of soybeans using electronic nose and HS-SPME-GC–MS. Food Chem. (2021) 340:127880. doi: 10.1016/j.foodchem.2020.127880, 32877847

[27] Wang R Yu S Huang Y Liu Y. Synthesis, high yield strategy and application of nisin: a review. Int J Food Sci Technol. (2023) 58:2829–41. doi: 10.1111/ijfs.16418

[28] Song X Qian L Zhang D Wang X Fu L Chen M. Effectiveness of differentiating mold levels in soybeans with electronic nose detection technology. Foods. (2024) 13:4064. doi: 10.3390/foods13244064, 39767006 PMC11675939

[29] Çelen T Anumudu C Miri T Onyeaka H Fernandez-Trillo P. Pathogen-responsive delivery of nisin. Food Hydrocoll. (2024) 154:110076. doi: 10.1016/j.foodhyd.2024.110076

[30] Ge S Chen Y Ding S Zhou H Jiang L Yi Y . Changes in volatile flavor compounds of peppers during hot air drying process based on headspace-gas chromatography–ion mobility spectrometry (HS-GC–IMS). J Sci Food Agric. (2020) 100:3087–98. doi: 10.1002/jsfa.10341, 32083310

[31] Su D He JJ Zhou YZ Li YL Zhou HJ. Aroma effects of key volatile compounds in Keemun black tea at different grades: HS-SPME–GC–MS, sensory evaluation, and chemometrics. Food Chem. (2022) 373:131587. doi: 10.1016/j.foodchem.2021.131587, 34838407

[32] Liu W Chen Y Liao R Zhao J Yang H Wang F. Authentication of the geographical origin of Guizhou green tea using stable isotope and mineral element signatures combined with chemometric analysis. Food Control. (2021) 125:107954. doi: 10.1016/j.foodcont.2021.107954

[33] Xiang Q Yuan X Yang A Li S Sun W Xu M . Effects of salt substitute and nisin on the flavor development of salt-reduced Pixian Douban (broad bean paste). J Food Sci. (2025) 90:e70169. doi: 10.1111/1750-3841.70169, 40183984

[34] Brandsma JB Brinkman J Wolkers-Rooijackers JCM van Swam I van Uitert K Zwietering MH . Pyruvate stimulates transamination of leucine into α-ketoisocaproic acid and supports 3-methylbutanal production by *Lactococcus Lactis*. J Appl Microbiol. (2024) 135:lxae257. doi: 10.1093/jambio/lxae257, 39380147

[35] Zeng L Fu Y-Q Liu Y-Y Huang J-S Chen J-X Yin J-F . Comparative analysis of different grades of Tieguanyin oolong tea based on metabolomics and sensory evaluation. LWT. (2023) 174:114423. doi: 10.1016/j.lwt.2023.114423

[36] Huang H Chen Y Hou Y Hong J Chen H Zhao D . Molecular sensomics combined with random forest model can reveal the evolution of flavor type of baijiu based on differential markers. Foods. (2024) 13:3034. doi: 10.3390/foods13193034, 39410069 PMC11476331

[37] Ai Y Kang N Montalbán-López M Wu X Li X Mu D. Enhanced stability, quality and flavor of bread through sourdough fermentation with nisin-secreting *Lactococcus lactis* Nz9700. Food Biosci. (2024) 62:105484. doi: 10.1016/j.fbio.2024.105484

[38] Prince A Sandhu P Ror P Dash E Sharma S Arakha M . Lipid-II independent antimicrobial mechanism of nisin depends on its crowding and degree of oligomerization. Sci Rep. (2016) 6:37908. doi: 10.1038/srep37908, 27897200 PMC5126574

[39] Banicod RJS Ntege W Njiru MN Abubakar WH Kanthenga HT Javaid A . Production and transformation of biogenic amines in different food products by the metabolic activity of the lactic acid bacteria. Int J Food Microbiol. (2024) 428:110996. doi: 10.1016/j.ijfoodmicro.2024.110996 39615409

