# Supplementary material for: Nisin improves the storage quality of refrigerated pre-packaged fermented soybean whey-tofu—food safety, texture, and flavor
Source: Front Nutr. 2026 Mar 30;13:1807620. doi: 10.3389/fnut.2026.1807620 (PMC13070952; doi:10.3389/fnut.2026.1807620)
Supplement: Supplementary file 1 [file Supplementary_file_1.docx]

**Table S1**

The electronic nose sensors corresponding to their primary response substances.

| Serial number | Sensor name | Primary response substances |
| --- | --- | --- |
| 1 | S1 | Aromatic compounds |
| 2 | S2 | Oxynitride |
| 3 | S3 | Ammonia |
| 4 | S4 | Hydrogenide |
| 5 | S5 | Olefes, arromas, and polar molecules |
| 6 | S6 | Alkanes |
| 7 | S7 | Sulfur compound |
| 8 | S8 | Alcohols, and partial aromatic compounds |
| 9 | S9 | Aryl hydrocarbon compounds, organic compounds of sulfur |
| 10 | S10 | Alkyl and aliphatic |

**Table S2**

Physicochemical results.

|  |  | 0 d | 4 d | 8 d | 12 d | 16 d | 20 d | 24 d | 28 d |
| --- | --- | --- | --- | --- | --- | --- | --- | --- | --- |
| WHC  （%） | CK | 82.64±0.53^Aa^ | 80.32±0.31^Bb^ | 78.11±0.57^Cc^ | 75.33±0.06^Dd^ | 72.33±0.41^Ce^ | 70.20±0.01^Cf^ | 68.93±0.18^Dg^ | 65.50±1.01^Dh^ |
|  | 0.5% | 82.64±0.53^Aa^ | 81.31±0.50^Ab^ | 78.63±0.32^Cc^ | 76.17±0.05^Cd^ | 74.43±0.21^Be^ | 72.16±0.40^Bf^ | 71.40±0.02^Cg^ | 70.58±0.28^Ch^ |
|  | 1.0% | 82.64±0.53^Aa^ | 81.53±0.45^Ab^ | 79.39±0.13^Bc^ | 76.96±0.08^Bd^ | 75.04±0.08^Ae^ | 73.44±0.45A^f^ | 72.30±0.43^Bg^ | 71.70±0.09^Bh^ |
|  | 2.0% | 82.64±0.53^Aa^ | 81.71±0.25^Ab^ | 80.11±0.15^Ac^ | 77.47±0.03^Ad^ | 75.21±0.08^Ae^ | 74.27±0.30^Af^ | 73.60±0.06^Ag^ | 72.01±0.15^Ah^ |
| Moisture  （%） | CK | 79.64±0.12^Ac^ | 83.94±0.12^Aa^ | 81.10±0.41^Ab^ | 79.16±0.26^Ac^ | 78.21±0.53^Ad^ | 76.03±0.30^Ae^ | 75.08±0.39^Af^ | 74.70±0.22^Af^ |
|  | 0.5% | 79.64±0.12^Ab^ | 82.95±0.11^Ba^ | 79.98±0.12^Bb^ | 77.44±0.50^Bc^ | 75.65±0.79^Bd^ | 73.67±0.29^Be^ | 72.28±0.23^Bf^ | 72.03±0.12^Bf^ |
|  | 1.0% | 79.64±0.12^Ab^ | 82.65±0.26^Ba^ | 79.09±0.50^Cc^ | 76.64±0.34^Bd^ | 75.38±0.36^Be^ | 73.01±0.21^Cf^ | 72.03±0.22^Bg^ | 71.23±0.15^Ch^ |
|  | 2.0% | 79.64±0.12^Ab^ | 82.32±0.13^Ca^ | 78.62±0.42^Cc^ | 76.27±1.07^Bd^ | 74.91±0.21^Be^ | 72.60±0.43^Cf^ | 71.77±0.06^Bg^ | 70.85±0.11^Dh^ |
| Total acidity | CK | 0.280±0.020^Ah^ | 0.316±0.020^Ag^ | 0.369±0.005^Af^ | 0.404±0.007^Ae^ | 0.487±0.004^Ad^ | 0.653±0.030^Ac^ | 0.875±0.020^Ab^ | 0.994±0.030^Aa^ |
|  | 0.5% | 0.280±0.020^Ah^ | 0.310±0.014^Ag^ | 0.331±0.009^Bf^ | 0.370±0.009^Be^ | 0.427±0.005^Bd^ | 0.491±0.003^Bc^ | 0.593±0.009^Bb^ | 0.719±0.028^Ba^ |
|  | 1.0% | 0.280±0.020^Ah^ | 0.299±0.006^Ag^ | 0.326±0.005^Bf^ | 0.365±0.013^Be^ | 0.401±0.020^Cd^ | 0.449±0.012^Cc^ | 0.551±0.015^Cb^ | 0.647±0.009^Ca^ |
|  | 2.0% | 0.280±0.020^Ah^ | 0.295±0.009^Ag^ | 0.322±0.008^Bf^ | 0.358±0.014^Be^ | 0.395±0.011^Cd^ | 0.443±0.008^Cc^ | 0.536±0.015^Cb^ | 0.635±0.007^Ca^ |
| pH | CK | 6.03±0.02^Ag^ | 6.00±0.02^Af^ | 5.95±0.01^Ah^ | 6.15±0.01^Ae^ | 6.17±0.01^Ad^ | 6.20±0.01^Ac^ | 6.22±0.01^Ab^ | 6.28±0.02^Aa^ |
|  | 0.5% | 6.03±0.02^Ac^ | 5.96±0.01^Be^ | 5.86±0.01^Bf^ | 5.98±0.01^Bd^ | 6.03±0.02^Bc^ | 6.07±0.01^Bb^ | 6.09±0.01^Bb^ | 6.14±0.01^Ba^ |
|  | 1.0% | 6.03±0.02^Ab^ | 5.91±0.01^Ce^ | 5.83±0.01^Cg^ | 5.88±0.01^Cf^ | 5.94±0.02^Cd^ | 5.99±0.01^Cc^ | 6.03±0.02^Cb^ | 6.09±0.02^Ca^ |
|  | 2.0% | 6.03±0.02^Ab^ | 5.90±0.01^Ce^ | 5.73±0.01^Dg^ | 5.85±0.01^Df^ | 5.90±0.02^De^ | 5.95±0.01^Dd^ | 5.99±0.02^Dc^ | 6.06±0.01^Da^ |
| colonies number  （CFU） | CK | 0.00±0.00^Ah^ | 2.44±0.11^Ag^ | 4.71±0.20^Af^ | 6.03±0.13^Ae^ | 7.07±0.10^Ad^ | 8.20±0.14^Ac^ | 9.59±0.13^Ab^ | 11.53±0.16^Aa^ |
|  | 0.5% | 0.00±0.00^Ah^ | 1.95±0.08^Bg^ | 2.76±0.10^Bf^ | 3.15±0.08^Be^ | 3.68±0.14^Bd^ | 3.99±0.07^Bc^ | 4.21±0.04^Bb^ | 4.80±0.05^Ba^ |
|  | 1.0% | 0.00±0.00^Ah^ | 1.75±0.08^Cg^ | 2.03±0.09^Cf^ | 2.65±0.09^Ce^ | 3.01±0.05^Cd^ | 3.45±0.08^Cc^ | 3.76±0.11^Cb^ | 4.17±0.08^Ca^ |
|  | 2.0% | 0.00±0.00^Ah^ | 1.64±0.09^Cg^ | 1.85±0.06^Cf^ | 2.49±0.05^Ce^ | 2.84±0.05^Cd^ | 3.25±0.09^Dc^ | 3.54±0.07^Db^ | 3.98±0.08^Ca^ |
| MDA  （mg/100g） | CK | 0.740±0.022^Ah^ | 1.226±0.064^Ag^ | 1.502±0.059^Af^ | 1.915±0.022^Ae^ | 2.648±0.012^Ad^ | 3.648±0.014^Ac^ | 3.927±0.227^Ab^ | 6.101±0.105^Aa^ |
|  | 0.5% | 0.740±0.022^Af^ | 1.165±0.070^Ae^ | 1.188±0.021^Be^ | 1.567±0.103^Bd^ | 1.784±0.293^Bc^ | 2.321±0.100^Bb^ | 2.336±0.062^Bb^ | 4.424±0.029^Ba^ |
|  | 1.0% | 0.740±0.022^Af^ | 1.066±0.030^Be^ | 1.160±0.016^Ce^ | 1.211±0.049^Ce^ | 1.433±0.0182^Bd^ | 2.039±0.022^Cc^ | 2.319±0.039^Bb^ | 4.017±0.094^Ca^ |
|  | 2.0% | 0.740±0.022^Ah^ | 0.991±0.003^Bg^ | 1.073±0.032^Cf^ | 1.157±0.008^Ce^ | 1.462±0.058^Bd^ | 1.928±0.062^Cc^ | 2.232±0.050^Bb^ | 3.914±0.007^Ba^ |
| TVB-N  （mg/100g） | CK | 1.15±0.11^Ah^ | 2.37±0.13^Ag^ | 4.03±0.09^Af^ | 6.13±0.11^Ae^ | 7.66±0.09^Ad^ | 8.58±0.14^Ac^ | 9.58±0.09^Ab^ | 10.41±0.06^Aa^ |
|  | 0.5% | 1.15±0.11^Ah^ | 1.81±0.07^Bg^ | 2.05±0.03^Bf^ | 2.38±0.01^Be^ | 3.14±0.04^Bd^ | 3.87±0.05^Bc^ | 4.70±0.05^Bb^ | 5.33±0.12^Ba^ |
|  | 1.0% | 1.15±0.11^Ah^ | 1.43±0.07^Cg^ | 1.85±0.05^Cf^ | 2.13±0.06^Ce^ | 2.47±0.09^Cd^ | 3.18±0.05^Cc^ | 3.62±0.05^Cb^ | 4.16±0.09^Ca^ |
|  | 2.0% | 1.15±0.11^Ah^ | 1.37±0.05^Cg^ | 1.79±0.06^Cf^ | 2.03±0.08^Ce^ | 2.40±0.08^Cd^ | 3.04±0.04^Cc^ | 3.51±0.02^Cb^ | 4.10±0.05^Ca^ |

**Note:** Lowercase letters indicate significant differences (*P* < 0.05) among storage time points within the same treatment group, while uppercase letters denote significant differences (*P* < 0.05) between treatment groups at the same storage time (28 d) for pre-packaged fermented soybean whey-based tofu.

**Table S3**

GC-IMS characteristic flavour ROVA values and their odour description.

| VOC | Odor threshold (ppm) |  | ROVA | | | | Verified odour description |
| --- | --- | --- | --- | --- | --- | --- | --- |
|  |  | group | 0d | 12d | 20d | 28d |  |
| Propionic acid hexyl ester | 0.02–0.05 | CK | 0.56 | 0.52 | 0.62 | 0.50 | Fruity, sweet aroma |
|  |  | nisin | 1.94 | 0.58 | 1.01 | 0.52 |  |
| 2-Ethylbutanal | 0.001 | CK | 35.47 | 24.58 | 42.77 | 28.10 | Green, fruity |
|  |  | nisin | 32.25 | 25.39 | 29.43 | 44.91 |  |
| 3-Methyl-3-buten-1-ol | - | CK | - | - | - | - | Green |
|  |  | nisin | - | - | - | - |  |
| Pyrrolidine | 0.05–0.2 | CK | 0.24 | 0.18 | 0.46 | 0.24 | Ammoniacal |
|  |  | nisin | 0.14 | 0.17 | 0.14 | 0.13 |  |
| (E)-2-Hexenal | 0.001 | CK | 74.28 | 67.25 | 76.19 | 63.70 | Strong green |
|  |  | nisin | 61.77 | 71.23 | 83.94 | 91.58 |  |
| Acetic acid ethyl ester | 0.005 | CK | 74.56 | 74.49 | 88.10 | 85.14 | sweet with slight wine-like notes |
|  |  | nisin | 72.42 | 95.01 | 90.64 | 73.94 |  |
| 2-Methyl pyrazine | 0.05 | CK | 1.04 | 0.47 | 0.48 | 1.07 | Nutty, roasted aroma |
|  |  | nisin | 0.28 | 0.15 | 0.33 | 0.39 |  |
| n-Propyl acetate | 0.02–0.1 | CK | 0.62 | 0.50 | 0.95 | 0.27 | Fruity |
|  |  | nisin | 0.57 | 4.19 | 1.74 | 0.84 |  |
| 2-Methylbutan-1-ol | 0.1–0.3 | CK | 0.52 | 0.38 | 0.51 | 0.31 | Alcoholic |
|  |  | nisin | 0.50 | 0.57 | 0.46 | 0.62 |  |
| 3-Penten-2-one | 0.01 | CK | 8.90 | 6.76 | 6.05 | 5.39 | Sweet |
|  |  | nisin | 8.23 | 5.20 | 9.49 | 9.00 |  |
| 4-Methylpentanol | - | CK | - | - | - | - | Alcoholic, soft aroma |
|  |  | nisin | - | - | - | - |  |
| Methyl isobutyl ketone | 0.05 | CK | 0.40 | 0.23 | 1.00 | 0.28 | minty with subtle fruity and ethereal notes |
|  |  | nisin | 0.45 | 2.16 | 1.15 | 0.51 |  |
| (E)-3-Hexen-1-ol | 0.001 | CK | 12.15 | 11.49 | 25.83 | 21.65 | leafy aroma like vegetables |
|  |  | nisin | 10.91 | 42.30 | 24.53 | 14.29 |  |
| Triethylamine | 0.02–0.1 | CK | 1.01 | 1.01 | 1.00 | 0.99 | Strong ammonia-like |
|  |  | nisin | 0.92 | 0.61 | 0.63 | 0.67 |  |
| 2-Methylbutanal | 0.001 | CK | 35.58 | 12.49 | 13.95 | 21.35 | fatty aroma like milk and cream |
|  |  | nisin | 31.67 | 35.19 | 13.59 | 33.47 |  |
| 2-Methyltetrahydrofuran-3-one | - | CK | - | - | - | - | Nutty, caramel-like |
|  |  | nisin | - | - | - | - |  |
| 2-ethyl furan | 0.005 | CK | 6.91 | 2.09 | 6.36 | 8.53 | nutty with subtle earthy notes |
|  |  | nisin | 5.85 | 6.21 | 3.49 | 1.81 |  |
| Ethyl 3-hydroxybutanoate | 0.05–0.2 | CK | 1.77 | 2.05 | 2.15 | 2.13 | Sweet, creamy |
|  |  | nisin | 1.48 | 1.90 | 2.03 | 2.13 |  |
| 2-Furaldehyde | 0.01–0.1 | CK | 1.39 | 1.53 | 1.04 | 3.06 | Roasted, nutty |
|  |  | nisin | 1.00 | 0.79 | 0.84 | 1.14 |  |
| Isovaleric acid | 0.1–0.5 | CK | 0.32 | 0.12 | 0.13 | 0.16 | Sweaty, cheesy |
|  |  | nisin | 0.20 | 0.16 | 0.21 | 0.07 |  |
| Butyl formate | 0.02–0.1 | CK | 4.33 | 1.70 | 2.21 | 3.16 | Fruity |
|  |  | nisin | 4.54 | 4.21 | 2.16 | 1.91 |  |
| Cyclohexane | - | CK | - | - | - | - | Slight gasoline-like |
|  |  | nisin | - | - | - | - |  |
| Pentanal | 0.001 | CK | 72.92 | 5.79 | 25.44 | 33.28 | Fatty aroma of fresh oils |
|  |  | nisin | 64.15 | 50.75 | 24.22 | 22.56 |  |
| 2-Ethyl-5-methylpyrazine | 0.01–0.05 | CK | 2.53 | 1.76 | 2.49 | 2.71 | Intense roasted |
|  |  | nisin | 1.65 | 3.14 | 1.77 | 0.68 |  |
| 1-Octene | - | CK | - | - | - | - | smell with faint fatty notes |
|  |  | nisin | - | - | - | - |  |
| Methyl 3-(methylthio)propanoate | 0.005 | CK | 9.26 | 4.14 | 6.36 | 7.05 | Sulfurous, meaty aroma |
|  |  | nisin | 9.26 | 7.07 | 5.44 | 3.15 |  |
| 2-Methylpyrazine | 0.1 | CK | 0.41 | 0.66 | 0.54 | 0.81 | Sulfurous, meaty aroma |
|  |  | nisin | 0.27 | 0.19 | 0.46 | 0.51 |  |
| 2-Methylpropyl butanoate | 0.05–0.1 | CK | 11.56 | 11.95 | 11.22 | 11.09 | Fruity, sweet |
|  |  | nisin | 11.45 | 11.86 | 12.01 | 10.54 |  |
| Ethyl butyrate | 0.01 | CK | 1.69 | 1.91 | 2.49 | 32.59 | Intense fruity aroma |
|  |  | nisin | 1.80 | 1.83 | 1.91 | 10.08 |  |
| 3-Methylbutanal | 0.001 | CK | 36.88 | 63.12 | 84.33 | 80.98 | Green, fatty aroma like cream |
|  |  | nisin | 41.22 | 100.00 | 63.82 | 82.56 |  |
| 3-Methyl-2-butenal | - | CK | - | - | - | - | Aldehydic with slight pungency |
|  |  | nisin | - | - | - | - |  |

**Table S4**

GC-MS characteristic flavour ROVA values and their odour description.

| **Apellation** | **Odor threshold/ppm** |  | **ROVA** | | | | **Verified odour description** |
| --- | --- | --- | --- | --- | --- | --- | --- |
|  |  | **group** | **0d** | **12d** | **20d** | **28d** |  |
| Vinyl amyl carbinol | 10.00 | CK | 9.53 | 13.89 | 12.54 | 7.05 | pungent, fresh, alcoholic |
|  |  | nisin | 13.13 | 12.98 | 45.91 | 6.59 |  |
| Octenol <2-trans-> | 20.00 | CK | - | - | - | - | Green apple aroma |
|  |  | nisin | - | - | 1.35 | 0.18 |  |
| Deca-(2E,4E)-dienal | 0.07~10 | CK | 0.88 | 0.87 | 0.55 | 10.29 | Fatty, greasy sensation |
|  |  | nisin | 0.88 | 0.51 | 0.99 | 3.66 |  |
| Hexanal <n-> | 4.1 | CK | 5.17 | 3.40 | 0.72 | 0.57 | Grassy, green freshness |
|  |  | nisin | 5.67 | 3.00 | 15.70 | 1.45 |  |
| Nonanal <n-> | 1~8 | CK | 3.92 | 3.70 | 2.20 | 1.88 | Fatty with citrus undertones |
|  |  | nisin | 4.17 | 2.72 | 12.28 | 2.18 |  |
| 2-Octenal, (E)- | 2.70 | CK | 15.42 | 2.39 | 2.60 | 4.77 | Fatty, grassy |
|  |  | nisin | 2.24 | 3.05 | 7.75 | 3.69 |  |
| Non-(2E)-enal | 0.08 | CK | 92.25 | 65.13 | 81.13 | 117.88 | Cucumber, grassy |
|  |  | nisin | 106.38 | 65.88 | 291.50 | 100.00 |  |
| Octanal <n-> | 0.70 | CK | 10.39 | 6.00 | 1.50 | - | Fatty, citrusy |
|  |  | nisin | 7.66 | 4.34 | 23.01 | 2.84 |  |
| Heptanal <n-> | 3.00 | CK | 5.77 | - | - | 2.97 | Rancid, sour |
|  |  | nisin | 4.75 | - | - | - |  |
| Hept-(2E)-enal | 13.00 | CK | - | 1.26 | 1.02 | 1.21 | Citrusy, fatty |
|  |  | nisin | - | 1.24 | - | 1.14 |  |
| Decanal <n-> | 0.1~2 | CK | - | - | - | 3.34 | Fatty, citrusy |
|  |  | nisin | - | - | - | - |  |
| Octen-3-one<1-> | 0.01 | CK | 812.00 | - | 804.00 | - | Mushroom-like, metallic |
|  |  | nisin | 780.00 | 688.00 | 4296.00 | - |  |
| Acetic acid, hexyl ester | 2.00 | CK | - | 2.31 | 4.40 | 1.06 | Fruity, fresh green |
|  |  | nisin | - | 2.39 | 2.11 | 0.49 |  |
| Hexanoic acid | 3.70 | CK | 1.04 | 1.74 | 0.66 | - | Sour, rancid, cheesy |
|  |  | nisin | 2.65 | 1.39 | 2.31 | 19.79 |  |
| Butyric acid | 6.80 | CK | - | - | - | 0.67 | Rancid, sour, buttery |
|  |  | nisin | - | - | - | - |  |
| Furan <2-pentyl-> | 6.00 | CK | 5.17 | 5.92 | 2.21 | - | Grassy |
|  |  | nisin | 4.85 | 3.58 | 16.77 | 1.64 |  |
